# Recent Trends in Polymer Matrix Solid Buoyancy Materials: A Review

**DOI:** 10.3390/polym16162307

**Published:** 2024-08-15

**Authors:** Xingcan Lu, Yu Li, Ze Chen, Shuaijie Li, Xiaoyan Wang, Qing Liu

**Affiliations:** Department of Chemistry and Materials, Naval University of Engineering, Wuhan 430033, China; xclu1816562907@163.com (X.L.); liyu62625252@163.com (Y.L.); lishuaijie_1@126.com (S.L.); a2954083292@163.com (X.W.); lq18895331186@163.com (Q.L.)

**Keywords:** buoyancy materials, polymers, hollow microspheres, interfacial modification

## Abstract

Polymer matrix solid buoyancy materials (PSBMs) have the advantages of low density, high strength, low cost, and low water absorption, and they are widely used in marine engineering fields. How to improve the performance of PSBMs further and adapt them to harsh marine environments has become a hot topic in current research. This paper provides a comprehensive summary of PSBM, detailing both the preparation methodologies and properties of single-component and multi-component PSBM. In this paper, relevant research is systematically summarized from two dimensions of matrix and filler, and the application of thermosetting resin and thermoplastic resin as a matrix in PSBM is introduced in detail, and the corresponding research on fillers such as hollow glass microspheres, fly ash, hollow ceramic spheres and hollow polymer microspheres are expounded. This paper aims to summarize the latest advancements in PSBM research, thereby providing insights into the current state of the field and guiding future investigations.

## 1. Introduction

With more than 70% of the Earth’s surface covered by oceans, the oceans contain mineral, energy and biological resources that far exceed those of the land [1]. Developing and utilizing the ocean has become a major focus for countries around the world [2]. However, exploring the ocean is not an easy task; for every 100 m increase in water depth, the pressure on an object increases by approximately 1.0 MPa, leading to a dramatic increase in the pressure that the equipment must withstand as the depth of the seawater increases. The development and utilization of marine resources cannot proceed without the support of advanced materials [3]. Solid buoyancy materials (SBMs) can provide buoyancy for submarines, unmanned underwater vehicles, and offshore equipment in different water depth environments, and they are one of the key materials that support the development of the marine industry.

**Figure 1:**
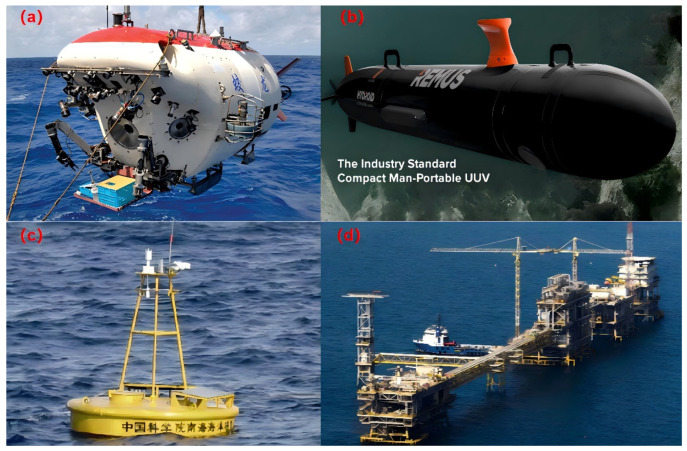
Different applications of SBM in the marine sector. (**a**) Jiaolong deep-submersible [10]; (**b**) REMUS UUV [11]; (**c**) Ocean buoys [12]; (**d**) Offshore oil platforms [13].

In the field of marine engineering, SBMs have garnered significant attention for their lightweight and high-buoyancy characteristics and are widely used in shipbuilding, ocean engineering structures, underwater detection equipment and marine resource exploitation. SBMs can reduce the weight of the hull, increase the load capacity, and reduce energy consumption. At the same time, the SBMs can provide the necessary buoyancy for the submarine to achieve deep diving and surfacing [4]. Among the offshore platforms and buoys, the SBMs ensure the stability and safety of the facility, enabling it to operate stably in extreme marine environments [5]. In addition, the application of SBM in underwater detection equipment facilitates the maintenance and management of the equipment, thereby improving the efficiency of underwater operations [6]. In the exploitation of marine resources, the riser floating body prepared by SBM establishes an oil and gas transportation channel from the submarine wellhead to the surface [7]. With the advances in marine resource development technology, the preparation and performance of SBM have become a research hotspot in the marine science and technology field [7,8,9]. Figure 1 shows a typical application example of SBM.

SBMs generally consist of a matrix and filler. The main types of matrices are polymers, ceramics and metals [14]. Fillers are generally low-density, high-strength hollow beads that act as density regulators in the material. Ceramic-matrix SBMs have low thermal conductivity and excellent high-temperature resistant; however, the open pores in the ceramic matrix affect the compressive strength of the buoyancy material. At the same time, when immersed in water, water easily enters the open pores of the material, and the material absorbs water at a high rate (30~40%), leading to changes in the buoyancy of the materials during use. Metal matrix SBM have excellent mechanical properties, high compressive strength, and are suitable for different water depth environments, but they are dense, limited in the buoyancy they can provide, and expensive to prepare. Compared to ceramic and metal matrix SBM, polymer matrix solid buoyancy materials (PSBM) are the first choice because of their low density, high strength, low water absorption, and low cost. In this paper, we provide a classification overview of PSBM based on different applicable water depths and composition structures and summarize the latest research progress in this field by domestic and foreign scholars in terms of improving the comprehensive performance of the material, focusing on the two dimensions of the matrix and filler. 

## 2. Classification and Overview of PSBM

Gas cavities in SBMs are the source of their buoyancy. The gas cavities in these materials can be either cells or various types of hollow beads. Based on the composition of the material, PSBM can be categorized into single-component, two-component, and three-component PSBM. Among them, single-component PSBMs have a large number of air-filled cells that provide buoyancy to the materials. Two-component PSBM and three-component PSBM, on the other hand, have gas cavities in various types of fillers with density regulating effects, which can be collectively referred to as multi-component PSBM and are the most widely used SBM in the deep-sea field [15,16,17,18].

### 2.1. Single-Component PSBM

Single-component PSBM, also known as polymer matrix syntactic foams (PFS), are traditional SBM with densities as low as 0.2 g/cm³ or less [19]. Single-component PSBMs form cells during the polymer molding stage, and their foaming processes are mainly divided into physical foaming and chemical foaming.

#### 2.1.1. Physical Foaming

Physical foaming technology mainly relies on Physical Blowing Agents (PBAs) such as CO_2_ and N_2_. These gases form a uniform dissolved state in the polymer matrix, which is subsequently induced by a pressure release or temperature change to form a foam material with a microporous structure. Physical foaming mainly includes batch foaming, injection molding foaming and extrusion foaming.

(a) Batch foaming

Batch foaming is a process for preparing polymer foams in batches under controlled conditions. This technique saturates the polymer in an autoclave with a physical blowing agent, such as CO_2_ or N_2_, and then induces the formation and growth of foam cells through a rapid increase in temperature or decrease in pressure [20]. Xu [21] et al. explored the effects of process parameters on the foam structure and volume expansion ratio using supercritical CO_2_ foamed polypropylene (PP) through a batch foaming process. A narrow suitable range of foaming temperatures and pressures was determined, and the foaming conditions were optimized to obtain a uniform foam structure. Urbanczyk [22] et al. discussed the cell morphology regulation of polystyrene-acrylonitrile (SAN)/clay nanocomposite foam prepared using supercritical CO_2_ as a foaming agent through a the batch foaming process. The results demonstrated the great flexibility of the supercritical CO_2_ batch foaming process in regulating the morphology of the foam cells.

The advantage of batch foaming is that it can precisely control the process of nucleation, cell growth, and cell structure stabilization, thus achieving a fine regulation of the foam microstructure. However, intermittent foaming usually faces the challenges of low processing efficiency and large-scale production difficulties; therefore, it is limited in industrial applications. Nevertheless, intermittent foaming is of great value in basic research and the development of new materials, especially in the preparation of foams with specific cellular structures.

(b) Injection molding foaming

**Figure 2:**
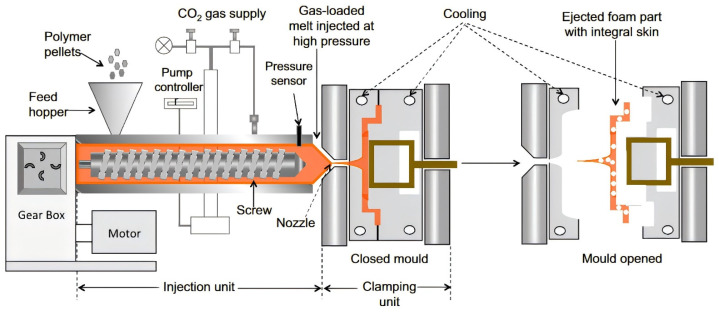
Schematic representation of injection molding foaming [25].

Injection molding foaming is an advanced manufacturing technology that combines traditional injection molding techniques with foam processing. In this technology, the polymer melt is mixed with a blowing agent in an injection molding machine and then injected into the mold to form a foam structure under the control of pressure and temperature [23]. Figure 2 is a schematic diagram of the injection molding foaming process. Yang [24] et al. prepared microporous polyether ether ketone (PEEK) by injection molding foaming, studied the effects of injection speed and injection volume and other process parameters on microporous PEEK, and optimized the preparation process to obtain two kinds of microporous PEEK, with the maximum weight loss rate of 17.29% and the maximum tensile strength of 74.13 MPa.

Injection molding foaming can achieve precise control of the foam shape and properties and prepare foam products with good mechanical properties and specific density [26]. In addition, this technology can reduce the amount of material used, shorten the production cycle, and improve production efficiency. However, the non-uniformity of the foam structure, surface quality problems and high requirements for mold design and processing accuracy also limit the application of injection molding foaming.

(c) Extrusion foaming

**Figure 3:**
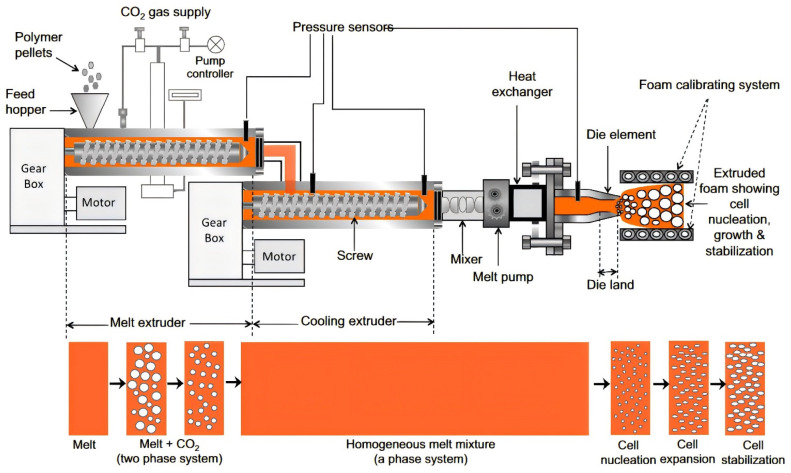
Schematic representation of extrusion foaming on a tandem-line [25].

Extrusion foaming is a continuous polymer foam preparation process that is widely used in industrial production. In this process, the polymer is melted in an extruder and mixed with a physical blowing agent to form a homogeneous polymer-gas solution. Then, through the geometric design and temperature control of the mold, the flow and foaming behavior of the polymer melt are adjusted, and finally, a foam product with a specific cross-section shape is formed. Figure 3 is a schematic of the industrial production line for extrusion foaming. Raje [27] et al. used CO_2_ and water as physical foaming agents to prepare polyether sulfone (PESU) foam materials by extrusion foaming, reducing the foaming temperature by about 100 ℃, and achieving large-scale continuous production.

The advantages of extrusion foaming technology are its high production efficiency and easy-to-scale production, which allows it to continuously produce foam materials. In addition, by adjusting the parameters of the extruder and mold design, the density, cell size, and distribution of the foam can be effectively controlled. However, during the extrusion foaming process, there may be issues, such as non-uniform cell size and difficulty in controlling the foam structure, which can affect the performance and application range of the final product.

#### 2.1.2. Chemical Foaming

**Figure 4:**
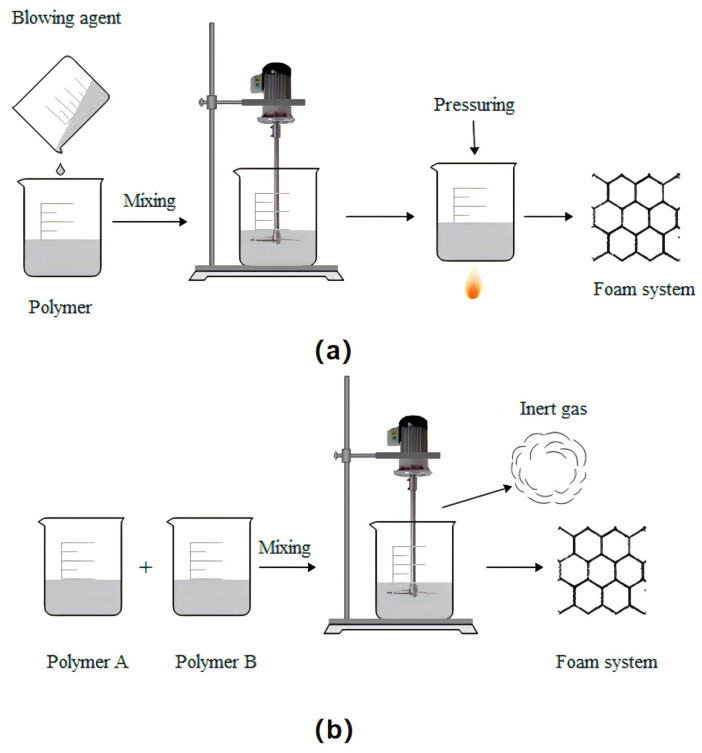
Two different schematics diagram of chemical foaming [28].

Chemical foaming is a method that utilizes chemical reactions to generate gas and form pores within a polymer. The two simple reaction processes of chemical foaming are shown in Figure 4: one is to add the foaming agent to the molten polymer and then decompose it to release the gas. The polymer is then foamed by pressure and heat (a), and the other is a chemical reaction between the two polymers to produce an inert gas, which forms the polymer (b). In addition to these two simple chemical foaming processes, chemical foaming materials are often prepared by extruders or injection molding machines in actual production. Most chemical foaming processes require the addition of chemical foaming agents. Therefore, according to the type of chemical foaming agent used, chemical foaming can be divided into organic chemical foaming and inorganic chemical foaming.

(a) Organic chemical foaming

Organic blowing agents are used in a wide range of applications. The decomposition reactions of organic blowing agents can be controlled such that their decomposition temperatures change within a given range. The most widely used chemical blowing agents are azodicarbonamide (blowing agent AC), dinitrosopentamethylene tetramine (blowing agent H) and 4,4′-oxydibenzenesulfonyl hydrazide (blowing agent OBSH).

Azodicarbonamide is an orange-yellow crystalline powder that colors the product in the case of incomplete decomposition and decomposes CO and NH_3_. Dinitrosopentamethylene tetramine is flammable, highly sensitive to acids and decomposes to produce CH_2_O and NH_3_. 4,4′-oxydibenzenesulfonyl hydrazide has a good dispersion in polymers, with a narrow decomposition temperature that is easy to control and fine bubbles that are homogeneous; it is widely used and is also called a universal foaming agent [29,30]. Xu [31] et al. discussed the direct extrusion foaming process of low-density polypropylene (PP) foam prepared by a single screw extrusion mechanism. The effects of the two chemical blowing agents on volume expansion behavior and cell morphology were investigated. The results showed that the use of azodicarbonamide as a blowing agent helped to achieve PP foam with greater volume expansion, while the use of the blowing agent EPIcor 882 produced a harder foam with a lower volume expansion ratio. At the same time, by precisely controlling the screw speed and mold temperature and other operating parameters, the goal of continuously producing low-density PP foam on an ordinary single screw extruder is successfully realized.

**Figure 5:**
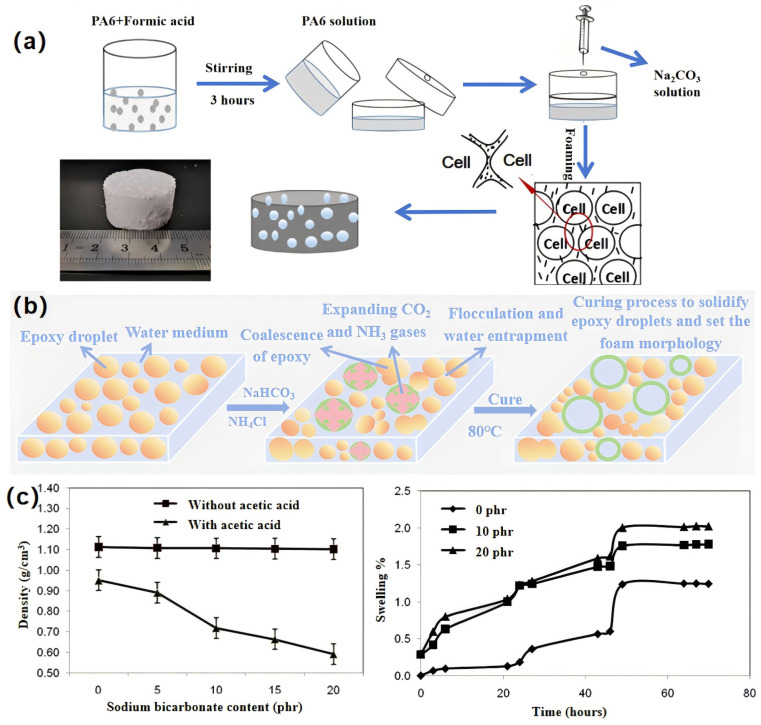
(**a**) Scheme of preparation procedure of porous PA materials [33]; (**b**) Polyamide–Epoxy aqueous emulsion foaming mechanism [34]; (**c**) Swelling percentage of epoxy foam without and with acetic acid at 20 phr of blowing agent content [35].

It is difficult for a single organic blowing agent to satisfy the requirements for blowing agents during material processing. The compounding of various organic blowing agents can achieve a balance of properties, such as exothermicity, dispersibility, decomposition temperature, and outgassing capacity. Zeng [32] et al. used azodicarbonamide/dinitrosopentamethylene tetramine (ACP-W) as a blowing agent to prepare an epoxy resin matrix single-component SBM with a density of 0.33 g/cm^3^ and a compression strength of 8.01 MPa (see Figure 5a), whose performance can meet the requirements for use in 800 m water depth environment.

Organic blowing agents have the advantages of good dispersion, stable gas output, uniform bubbles, etc. At the same time, the decomposition temperature is lower, and it is easy to control the release of gas. However, the decomposition temperature of organic blowing agents, such as ACP-W, is high, and the blowing agents need to decompose and release gas at 130 °C. A high reaction temperature tends to promote the curing of the resin, and the rapid change in viscosity is not conducive to the formation of a uniform cell structure. There are two main processes for single-component PSBM preparation: chemical foaming and polymer curing. Temperature is an important process parameter in these two processes, and how to coordinate the selection of the appropriate temperature is the key to the material properties.

(b) Inorganic chemical foaming

In practical production, inorganic blowing agents are used more frequently, mainly bicarbonate, carbonate, nitrite, and hydrogen peroxide. The inorganic blowing agents are relatively low cost and suitable for large-scale industrial production. Wang [33] et al. injected aqueous sodium carbonate solution into formic acid-impregnated Nylon-6 pellets, which neutralized and reacted to produce a large amount of CO_2._ Forming a cell structure to prepare polyamide matrix chemically blowing single-component buoyancy material with a density of 0.1 g/cm^3^ or less (the preparation process is shown in Figure 5b).

Inorganic foaming agent reaction conditions are simple, which is conducive to improving efficiency and reducing costs. In most inorganic foaming reaction processes, heat absorption can easily control the temperature, and the gas released by the reaction (CO_2_, etc.) is safe. However, the dispersion of the inorganic blowing agent in the polymer matrix is poor, and the distribution of cells is not uniform. The detailed mechanism of gas foaming needs to be clarified to facilitate the control of the distribution and size of the cell. Du [34] et al. successively added ammonium chloride and sodium bicarbonate to polyamide–epoxy resin aqueous emulsions and utilized the CO_2_ and NH_3_ expansion gases generated by the reaction to form gas bubbles. As shown in Figure 5c, the foaming mechanism is as follows: the epoxy phase is dispersed in the aqueous phase, and the foaming reaction of sodium bicarbonate and ammonium chloride proceeds at room temperature, producing carbon dioxide (CO_2_) and ammonia (NH_3_), which are trapped in the epoxy emulsion. During the reaction, the gases expand, forcing the epoxy droplets to move to one side until they flocculate and partially agglomerate, and the water in the epoxy emulsion evaporates during curing to form pore bubbles.

Single-component PSBM prepared with inorganic blowing agents have the advantages of low density and a simple fabrication process, but the microstructure of the gas cavities leads to the materials being weak. Hamad [35] et al. used sodium bicarbonate as a blowing agent to prepare an epoxy chemically blown single-component buoyancy material. The results of the study are shown in Figure 5c; although the addition of a certain amount of acetic acid significantly increases the pore size, it also leads to a decrease in material density and a significant reduction in mechanical properties.

#### 2.1.3. Summary of This Section

The single-component PSBMs form a cellular structure in high polymer using physical or chemical foaming technology to achieve lightweight and the desired buoyancy characteristics.

Batch foaming, injection molding foaming and extrusion foaming are the three main processes of physical foaming. The batch foaming process can precisely regulate the nucleation and growth of foam cells under controlled conditions, but it is limited by processing efficiency and the difficulty of large-scale production. Injection molding foaming combines injection molding and foaming technology to achieve precise control of foam shape and properties, despite challenges such as the uneven structure of the foam. Extrusion foaming is a continuous production process through mold design and temperature control to regulate the flow and foaming behavior of polymer melt to achieve high production efficiency and large-scale production; however, there is a problem of cell size heterogeneity.

Chemical foaming is the formation of cells in a polymer through a chemical reaction and is divided into organic chemical foaming and inorganic chemical foaming. Organic chemical blowing agents, such as azodicarbonamide, have the advantages of good dispersion and stable gas output; however, the decomposition temperature is high, which may affect the uniformity of the bubble structure. Inorganic chemical blowing agents, such as bicarbonate and carbonate, have low cost and are suitable for large-scale production; however, poor dispersion may lead to an uneven distribution of cells.

The preparation technology of single-component PSBM involves two main methods: physical foaming and chemical foaming, each of which has its advantages and limitations. A thorough understanding of the foaming mechanism, the optimization of the foaming agent selection, and process parameter regulation is of great significance in achieving a balance of material properties and expanding the application field.

Single-component PSBMs do not add density regulator media, such as hollow microspheres and rely on the formation of cells from the gas generated during the synthesis reaction to provide buoyancy. The gas cavity microstructure leads to low compressive strength, poor stability, and high water absorption rate of a single-component PSBM under medium- and deep-sea conditions and is prone to rupture and seepage, which limits its practical application scope. In practical engineering applications, single-component PSBMs are suitable only for shallow sea areas below 600 m.

### 2.2. Multi-Component PSBM

Two-component PSBM and three-component PSBM, collectively referred to as multi-component PSBM or polymer matrix syntactic foams (PFS), are based on a resin matrix to which fillers with low-density and high compressive strength properties are added to provide higher mechanical strength, durability, and stability to meet the related deep-sea field’s demand for solid buoyancy materials in relevant deep-sea fields [36,37,38,39,40]. The buoyancy sources in multi-component PSBM are generally hollow microspheres, mostly hollow glass microspheres (HGMS), but also other low-density fillers, such as hollow ceramic spheres. While these fillers provide more effective support for the buoyant materials, they also inevitably increase the density of the materials.

#### 2.2.1. Two-Component PSBM

Since the 1960s, the United States, Japan, Russia, and other countries have begun to develop SBMs that are suitable for deep-sea environments. In 1964, the USS Alvin, funded by the U.S. Navy was the first to use a two-component SBM, which consists of hollow glass microspheres filled with epoxy resin, in the design and fabrication of deep-sea manned submersibles. At present, two-component PSBMs have been widely used in scientific research, production, and military industries [41].

A two-component PSBM typically consists of a lightweight component and a continuous, denser matrix component. A lightweight component typically consists of HGMS or other lightweight fillers that provide the desired buoyancy. The matrix component consists of a high-performance polymer resin that forms a continuous network to ensure the mechanical strength and structural integrity of the material.

**Figure 6:**
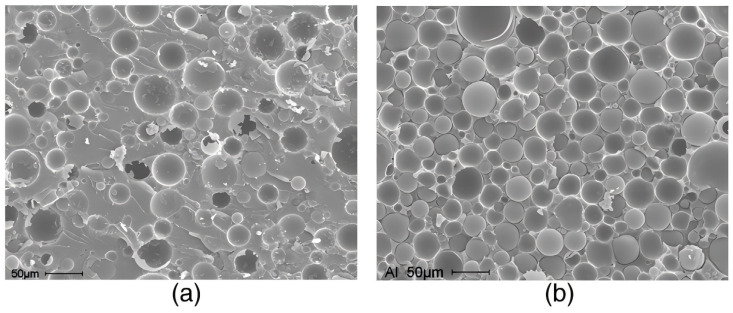
Scanning electron micrographs of the vinyl ester matrix syntactic foams containing (**a**) 30 and (**b**) 60 vol. % glass microsphere s of 220 kg/m^3^ density [44].

A large number of researchers have investigated the properties of two-component PSBM. Gupta [42,43] et al. prepared a two-component PSBM using different HGMS and investigated its properties. The experimental results showed that the compressive modulus, compressive strength, and total energy uptake of this PSBM could be modulated by choosing a suitable microsphere type and volume fraction. The compressive modulus and compressive strength increase with decreasing HGMS inner diameter for the same volume fraction. The compressive modulus and compressive strength decrease with increasing volume fraction for the same HGMS type. The compressive strength and modulus of the PSBM, on the other hand, are closely related to the strength and modulus of the weakest layer in its structure. The strength and modulus depend on the strength and modulus of the weakest layer in its structure. In addition, Gupta’s study also found that the area under the stress–strain curve of PSBM material is 300–500% higher than that of ordinary composite foam, which indicates that the two-component material is able to absorb more energy than the single-component materials. Poveda [44] prepared vinyl ester matrix composite foam based on Gupta’s material (its SEM image is shown in Figure 6). The effect of wall thickness and the volume content of hollow glass microspheres on the macroscopic Poisson’s ratio of the material was investigated. The results showed that the Poisson’s ratio of the material decreased with the increase of microsphere content. It was also found that the wall thickness of microspheres has little effect on the Poisson’s ratio of the material in the case of small microsphere content.

The two-component PSBM can maintain low water absorption even when immersed in seawater for a long period of time, avoiding the high intensity of performance degradation caused by water absorption. However, according to the “parametric theory of random particle stacking” [45], the maximum volume fraction of hollow microspheres in two-component PSBM is about 60–70%. Relevant research results show that [46,47] the minimum density of the two-component PSBM that can be achieved is about 0.4–0.5 g/cm^3^. Due to the limitations of density and cost, a two-component PSBM is suitable for ultra-deepwater, and it is difficult to further reduce the density of PSBM by continuing to add the hollow microspheres, so it is necessary to try other technological paths.

#### 2.2.2. Three-Component PSBM

To further reduce the densities of PSBM, researchers have attempted to prepare a three-component PSBM by adding large-size lightweight hollow materials, such as hollow ceramic spheres, to the two-component PSBM [48]. Cui [49] et al. proposed a new method of adding hollow ceramic spheres to the large-size SBM in order to reduce the density from 0.7 g/cm^3^ to 0.64 g/cm^3^. A high filling stacking factor of hollow ceramic spheres can be realized by adjusting the different particle sizes of the hollow ceramic spheres. Wu [50] et al. prepared three-component materials with different volume fractions by using epoxy resin, graphite-reinforced hollow epoxy spheres (GR-HEMS) and HGMS. The results of the study are shown in Table 1 and Table 2. As the volume fraction of GR-HEMS increases, the density of the material decreases, and the compressive strength decreases; the wall thickness of GR-HEMS increases, the density increases, and the strength of the material increases. When the number of prepared layers of GR-HEMS is increased from 1 to 3, the compressive strength of the three-component PSBM is increased from 16.0 MPa to 25.2 MPa. The three-component PSBM achieves a low density with a decrease in compressive strength resistance, and it is usually used in the mid-depth sea field.

In practical engineering applications, larger spheres may implode in service due to overpressurization, and the potential energy in the flow field generates shock waves. Shock waves further lead to the implosion of the surrounding sphere, leading the buoyancy system to catastrophic failure. For example, the U.S. “Poseidon” hybrid submersible was lost due to the implosion of the buoyancy material during full-depth operation [51]. In order to avoid the implosion of centimeter-sized hollow spheres under high-pressure conditions in deep-sea environments, researchers have attempted to reinforce the matrix. Jiang [52] et al. prepared HGMS-reinforced epoxy hollow spheres (HGMS-EHS) and mixed HGMS, HGMS-EHS, epoxy resin, glass fibers (GF), and curing agent to prepare glass fiber-reinforced epoxy composite foam material (GF-FSF), which is a composite material that can be used as a buoyancy material in the deep-sea environment. The experimental results showed that by increasing the number of prepared layers of HGMS-EHS, the strength of HGMS, and decreasing the stacking fraction and particle size of HGMS-EHS, the compressive strength of GF-ESF increased, but the density also increased significantly.

Three-component PSBM containing large-sized hollow spheres have a lower density compared to two-component PSBM, but at the same time,, their compressive strength is lower. For example, three-component PSBM containing high-strength ceramic hollow spheres have high strength and low density, making them ideal SBM for large depths. However, ceramic hollow spheres may face the problem of implosion under high pressure. How to increase the strength while reducing the density of the hollow microspheres to enhance the integrity of the material is a major challenge in the research neighborhood of PSBM.

#### 2.2.3. Modeling of Multi-Component PSBM Performance

Since PSBMs are commonly used in marine environments, compressive strength is one of the most important performance indicators. Therefore, researchers have also studied the compressive properties of PSBM. To simulate the synergistic effect of matrix and filler under high-pressure conditions on the seabed, predictive models are often developed with the help of simulation and modeling software to study the material properties.

De Pascalis [53] et al. developed a model to predict the pressure-relative volume change, revealing that the macroscopic mechanical behavior of two-component composite buoyant materials is strongly dependent on the microstructure of HGMS in terms of distribution, stiffness and volume fraction. Chen [54] et al. established a body-centered cubic cell model composed of HGMS and epoxy resin matrix and numerically simulated the model with different microsphere volume fractions and different wall thickness compositions with the help of ANSYS finite element software. Wang [55] and others showed that when the HGMS strength is low, material failure is mainly caused by HGMS crushing, while when the HGMS strength is high, material failure is mainly caused by the peeling of the epoxy matrix interface. However, due to the differences in the internal microstructure of the material, different failure modes will occur under compressive loading.

Modeling using finite element software has a significant effect on both predicting material properties and guiding material preparation. However, in practice, buoyancy materials are subjected to a much harsher environment, and consideration needs to be given to incorporating the combined effects of other external conditions, such as temperature and salinity.

#### 2.2.4. Influence of Preparation Process on the Performance of Multi-Component PSBM

The casting molding method, vacuum molding method and compression molding method are the three main molding techniques used in the preparation of multi-component PSBM. These methods regulate the buoyancy properties, mechanical strength, and durability of materials by controlling their density, pore structure, and packing distribution. Different molding processes are decisive for the microstructure and macroscopic properties of the materials, and the effects of these preparation processes on the comprehensive properties of the materials will be discussed in detail below.

(a) Casting molding method

The casting molding method is the main method for preparing PSBM in the laboratory [56,57]. This method mixes a polymer matrix with lightweight fillers and casts them into a mold to achieve uniform filling and curing of the material. This results in buoyancy materials with good surface quality and a uniform microstructure. Advantages include ease of handling, customized production, and high material utilization. However, this method also has limitations such as long curing time, difficulty in controlling dimensional stability, and low degree of automation. In the actual preparation process, the shear force generated by stirring tends to break hollow fillers, such as HGMS. The proportion of broken particles in the matrix resin is too large, and the density of the material increases. At the same time, the irregular size distribution of the broken particles in the matrix forms a stress concentration, reducing the strength of the material. In addition, for some high-performance materials, the casting molding method is difficult to meet, especially in terms of their specific processing requirements.

(b) Vacuum molding method

**Figure 7:**
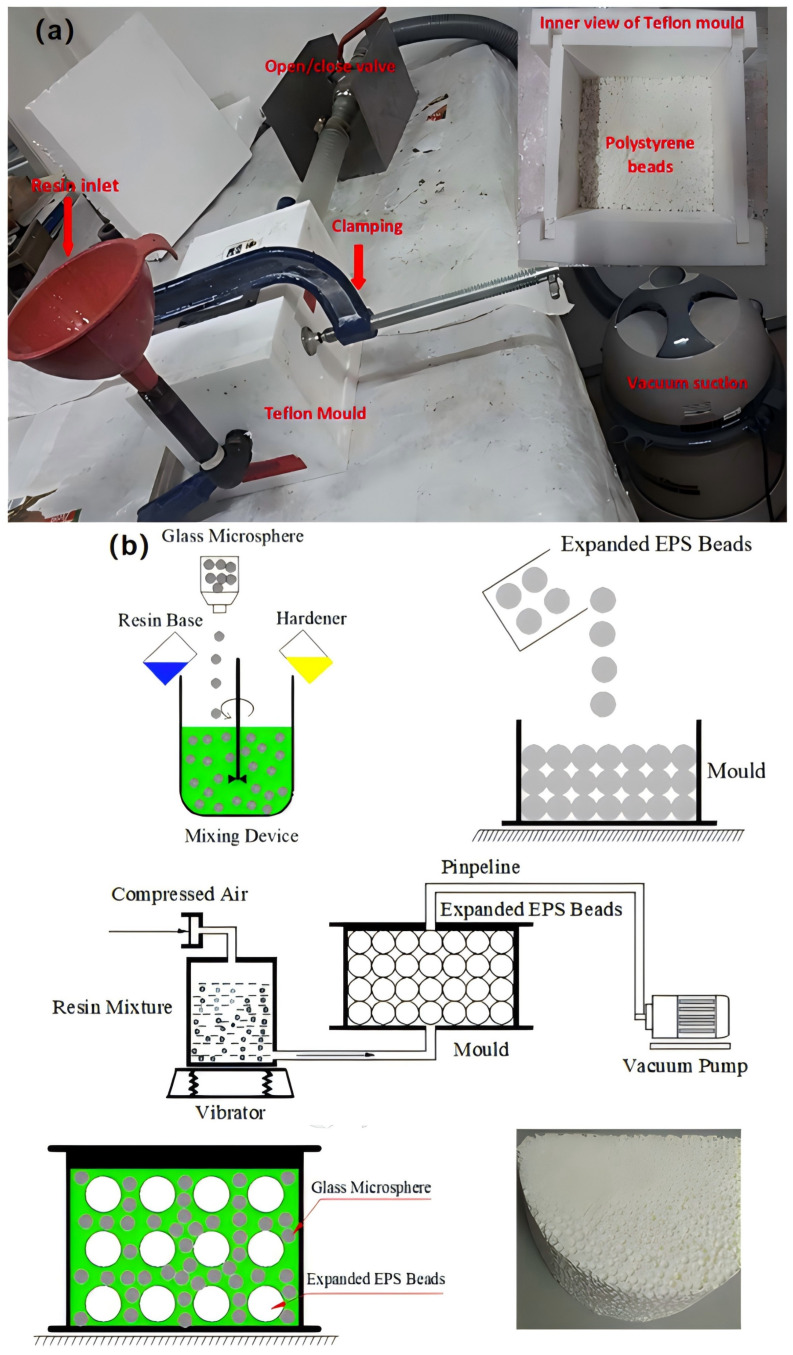
(**a**)Vacuum Assisted Mold Technique (VAMT) apparatus set-up [58]; (**b**)Schematics of Pressure molding method [59].

In order to solve the problems of the casting molding method, researchers have invented a vacuum-forming method. The vacuum-forming method is mainly used to completely mix the resin with lightweight fillers by creating a negative-pressure environment, after which the resin is cross-linked and cured to form composite foam materials. Afolabi [58] et al. designed a vacuum-forming device (see Figure 7a) in which the hollow microspheres were filled into the mold, and the resin matrix was gradually poured into it to completely infiltrate the hollow microspheres while the vacuum device was activated to maintain the vacuum environment inside the mold. The vacuum environment allows the matrix and filler to directly combine closely, and the strength of the material is significantly improved. However, this method relies too much on the vacuum pressure difference to realize the material densification and curing and requires a long time of vacuum treatment to achieve the ideal densification, resulting in lower production efficiency. In addition, the vacuum-forming method requires high sealing of equipment and molds, which increases manufacturing costs and maintenance difficulties. In some cases, the resin flow and curing process under vacuum conditions may be inhomogeneous, affecting the consistency and performance of the material.

(c) Compression molding method

The researchers adapted the vacuum molding method, which is the compression molding method, to tightly combine the resin with the lightweight filler through positive pressure, and then crosslink and cure the resin to form a composite material. Figure 7b shows the schematic of the route used by Yu [59] et al. to prepare epoxy resin matrix composite foams using the compression molding method: firstly, the epoxy resin matrix was mixed with HGMS and the gases in the mixture were degassed by vacuum; then, the expanded polystyrene beads (EPS) were placed into a mold and compacted with a lid, and a hose was used to connect the compressed air, the resin mixture, a shaker, the mold, and a vacuum pump. The resin mixture is pressed into a mold filled with EPS beads, and the product is obtained after curing and demolding.

Compared to the vacuum molding method, the compression molding method has lower equipment requirements and better product performance [60]. The compression molding method realizes the densification of the material through pressure, which enhances the mechanical properties and structural stability and is suitable for mass production. However, the compression process is prone to cause uneven internal stresses in the material, which affects the homogeneity of the material; therefore, the optimization of the process parameters is crucial.

#### 2.2.5. Summary of This Section

Currently, multi-component PSBMs are widely studied and applied. The results of a large number of related studies have revealed the laws of material property changes, and the relationship between material properties and microstructure has been predicted through modeling.

At present, PSBMs are mainly developed in the following directions: First, the resin matrix properties of buoyancy materials play a key role in the overall performance, and high-performance PSBM can be prepared by improving the matrix properties; secondly, the comprehensive matching of the strength and density of the buoyancy materials is the core performance index, and it is necessary to reduce the density under the premise of ensuring the strength to prepare buoyancy materials with more superior performance; the interfacial connection performance and interfacial compatibility performance between the filler and the matrix seriously affect the comprehensive performance of the PSBM. The use of hollow polymer microspheres(HPMS) and interfacial modification are adopted to improve them in order to enhance the comprehensive performance of buoyancy materials.

## 3. Polymer Matrix

The overall performance of the PSBM is related to the properties of the polymer matrix and reinforcing phase. The resin matrix, as the carrier of each hollow structural unit, is responsible for equalizing the material load to improve the overall strength of the material and is required to have low density, high strength, low water absorption, and chemical stability. In addition, in order to adapt to the preparation process of PSBM, the polymer matrix should have a low viscosity, long application period, low exothermic curing process, low shrinkage of the curing reaction, and good compatibility with the hollow beads. Selecting an appropriate polymer matrix based on the operational conditions is crucial for the advancement of PSBM. Table 3 shows the properties of several commonly used polymer resins.

Polymer matrices currently used in most PSBM include mainly thermoplastic and thermosetting resins. Thermoplastic resins include polyethylene [61], polypropylene [62,63,64,65], polyamide [66,67,68], vinyl resins [69], etc. Thermosetting resins applied to PSBM include epoxy resins [70,71,72,73,74,75], polyurethane [76,77,78,79], and phenolic resins [80,81,82,83,84], among others. Due to the differences in the structures and properties of thermoplastic resins and thermosetting resins, their preparation processes are also quite different. In the following paper, the research on the application of thermoplastic and thermosetting resin matrixes in PSBM is described, respectively.

### 3.1. Thermoplastic Resins

The melting and curing processes of thermoplastic resins are reversible, which means that the materials can be repeatedly heated to melt and cooled to cure without changing their chemical structure. This property makes them easy to mold during processing into a PSBM and can be machined into desired shapes. At the same time, the excellent toughness of thermoplastic resins prevents complete destruction by ductile fracture under high water pressure. However, research on the thermoplastic resin matrix SBM is relatively limited. The following section introduces the properties of several common thermoplastic resins.

#### 3.1.1. High-Density Polyethylene (HDPE)

The density of HDPE is less than 1 g/cm^3,^ and its use as a matrix can effectively reduce the density of the PSBM. Jayavardhan [85] et al. prepared HGMS/HDPE matrix SBM by compression molding method and investigated the relationship between filler content and mechanical properties such as compression strength and tensile strength.

However, the compressive strength of HDPE makes it difficult to meet the harsh conditions of high pressure and high temperature in the deep sea, so researchers modified HDPE in the hope of enhancing its performance as matrix. Bharath Kumar [86] et al. functionalized HDPE with dibutyl maleate to prepare HDPE matrix composite foam materials. The results of the study showed that the density of the material was reduced by the addition of hollow glass microspheres. Cosse [87] et al. used grafted maleic anhydride of polyethylene as a matrix to prepare PSBM possessing good mechanical properties.

HDPE exhibits excellent buoyancy and durability as a PSBM matrix with a low density and good chemical stability. However, its low heat deflection temperature and relatively poor pressure resistance limit its application in extreme environments. In addition, long-term UV exposure may lead to deterioration, requiring the addition of stabilizers to enhance weather ability. Despite these limitations, with proper material design and modification, HDPE can be a cost-effective matrix option that deserves more attention.

#### 3.1.2. Polypropylene (PP)

PP is a lightweight, high-strength, and chemically resistant thermoplastic polymer that is widely used in several industrial applications due to its excellent processability and cost-effectiveness. Doumbia [88,89] et al. prepared lightweight and high-strength composite foams by incorporating different HGMs into polypropylene resins. Considering the thermoplastic resins properties of polypropylene resins, a compression molding method was used for the preparation.

Chariere [90] et al. investigated in detail the degradation behavior of HGMs blended systems with high-impact polypropylene (HIPP) and their effect on the composite properties. The tolerances of different HGMS during melt processing were experimentally evaluated, and their effects on the density and properties of HIPP were analyzed. It was found that a specific HGMS could effectively resist melt-processing and significantly reduce polymer density while maintaining good mechanical properties. The results of the study are important for the development of lightweight and high-performance thermoplastic materials.

**Figure 8:**
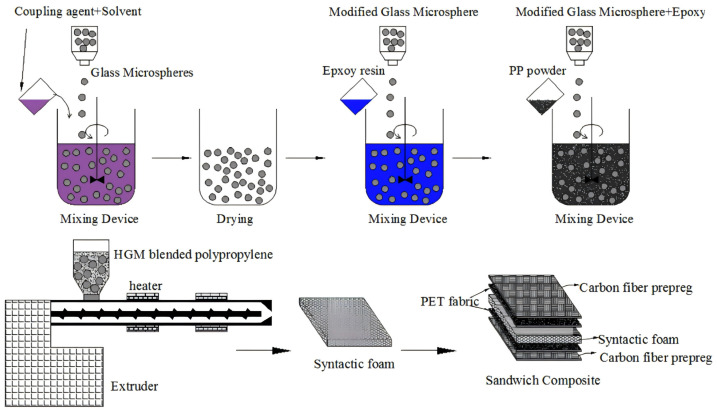
The experimental procedures for producing PP matrix syntactic foam and sandwich composites [91].

Qi [91] et al. used a twin-screw extruder to prepare thermoplastic and thermosetting composite foams. Figure 8 shows the process of preparing sandwich composites with the two composite foams as the core material and carbon fiber as the surface layer. The results show that the mechanical properties of the thermoplastic resin (PP) matrix composite foam are inferior to those of the thermosetting resin (epoxy resin) matrix composite foam. In order to improve the mechanical properties of the former, epoxy resin was mixed with PP to prepare a hybrid matrix; however, the interfacial compatibility problem resulted in defective materials.

#### 3.1.3. Poly (Methyl Methacrylate) (PMMA)

PMMA is a thermoplastic polymer with excellent clarity and superior weatherability, and is known for its light weight, chemical stability, and biocompatibility. PMMA shows potential for a wide range of applications in many industries, particularly in the field of high-performance SBM, where its unique physical and chemical properties make it ideal for deep-sea engineering and other marine applications.

Lamm [92] et al. proposed an innovative strategy for preparing thermoplastic composite foams based on PMMA using HGMS and expandable thermoplastic microbeads (EMS). The method systematically optimized the addition amounts of HGMS and EMS, leading to the fabrication of a lightweight, highly porous, and low thermal conductivity foam structure. It was shown that an additive amount that is too high leads to a decrease in thermal insulation properties and mechanical stability. The material exhibits lightweight and high-strength properties and adjustable thermal insulation, with the potential for use in high-pressure and high-temperature environments.

**Figure 9:**
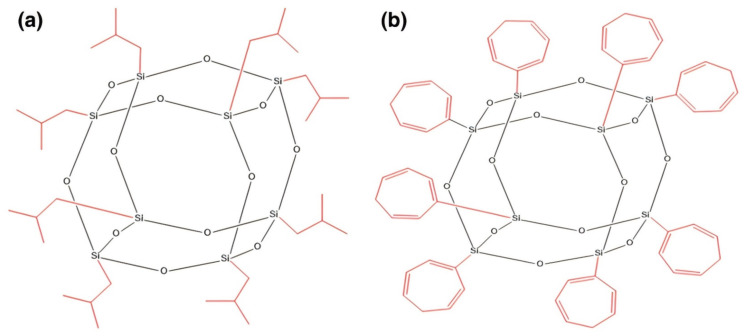
Molecular structure of (**a**) octaisobutyl POSS and (**b**) octaphenyl POSS [93].

As can be seen from Table 1, the mechanical properties of thermoplastic resins may not be as good as those of thermosetting resins; therefore, researchers have chemically modified thermoplastic resins to enhance their properties. Ozkutlu [93] et al. used two polyhedral oligomeric silsesquioxanes to enhance the modification of PMMA (Figure 9 shows the structural schematic of the two polyhedral oligomeric silsesquioxanes) with the addition of HGMS to prepare syntactic foams. The results showed that the specific flexural strength of PMMA modified with POSS increased by 18%, and the improved performance of the polymer matrix gave the syntactic foams more excellent performance.

PMMA offers excellent transparency and luminosity as well as good mechanical strength and toughness. PMMA’s low density and adjustable mechanical properties expand its potential as a buoyancy material by filling it with lightweight materials. In addition, its environmental stability provides an added advantage for specific marine sector applications. Despite the need to consider its long-term water resistance and cost-effectiveness, PMMA’s combined properties show potential as a matrix for PSBM.

#### 3.1.4. Summary of This Section

Thermoplastic resins can be repeatedly heated, melted and cooled to solidify, with easy processing and recyclability, as well as good toughness, which can reduce the crack extension phenomenon in the material; these characteristics in the buoyancy material field have attracted widespread attention.

However, its use as a matrix for PSBM still has the following limitations: 1. The bottom water temperature in deep-sea local areas is very high; for example, in hydrothermal vents on mid-ocean ridges and a small area, the temperature can be as high as 300–400 °C, and thermoplastic resins are easy to soften or melt at high temperatures, which restricts its application in extreme environments; 2. Insufficient mechanical strength and rigidity affect the carrying capacity and durability of PSBM; 3. The sensitivity of some thermoplastic resins to chemicals limits their applications in specific chemical environments; 4. Poor resistance to hydrolysis and microbial erosion of thermoplastic resins; 5. Inadequate interfacial bonding, which may affect overall material performance. In order to improve the applicability of PSBM, the above problems should be studied to prepare thermoplastic resins with excellent performance.

### 3.2. Thermosetting Resins

Thermosetting resins, a class of polymers that form an irreversible three-dimensional network structure upon curing, are widely used in the fabrication of PSBM because of their exceptional heat resistance, excellent mechanical properties, and good chemical stability. The chemical cross-linking reactions that occur during the curing process of these resins provide materials with an extremely high degree of structural integrity and durability, allowing them to maintain their performance even when subjected to complex stresses of the marine environment over long periods of time. In addition, thermosetting resins have a tunable curing process that facilitates precise processing and molding, and the cured materials typically have low water absorption and good hydrolysis resistance, which is critical for ensuring the buoyancy maintenance of buoyancy materials in underwater applications. Therefore, thermosetting resins, as matrix materials, provide the necessary stability and reliability for PSBM and are the main reason for their use as mainstream resin matrices for PSBM. The following section describes recent research advances in several thermosetting resin matrices and curing processes commonly used for PSBM.

#### 3.2.1. Phenolic Resin (PF)

**Figure 10:**
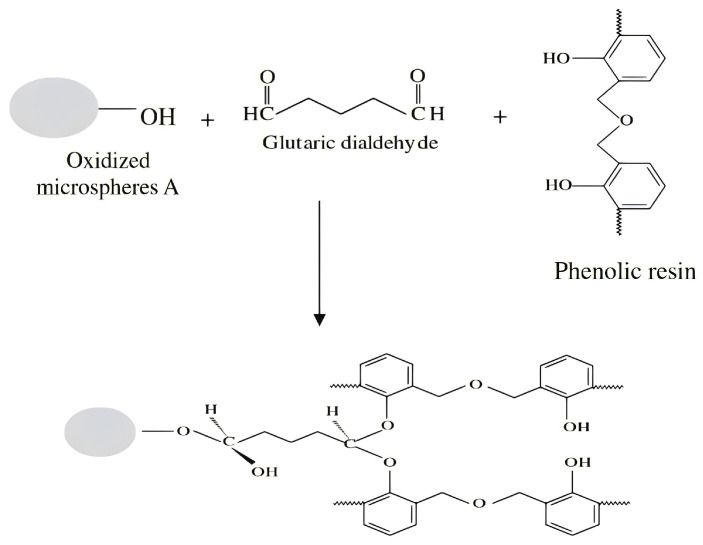
Schematic process of chemical reaction between the oxidized hollow carbon microsphere and phenolic resin [97].

Phenolic resins are favored for marine engineering and buoyancy material applications due to their excellent thermal stability, superior mechanical strength, and good chemical resistance. Their thermosetting network structure provides a material with dimensional stability at extreme temperatures [94,95], which is crucial for environments with large temperature fluctuations, such as the deep sea. In addition, the low water absorption of phenolic resins helps maintain the long-term performance and durability of buoyant materials. Due to their excellent properties, syntactic foams derived from phenolic resins are used in a variety of applications, especially in lightweight structures, aerospace facilities, and buoyancy applications [96]. Zhang [97] et al. prepared syntactic foams by incorporating hollow carbon microspheres treated with a coupling agent into a phenolic resin matrix. Figure 10 illustrates the reaction scheme between the hollow microspheres, coupling agents, and phenolic resin.

**Figure 11:**
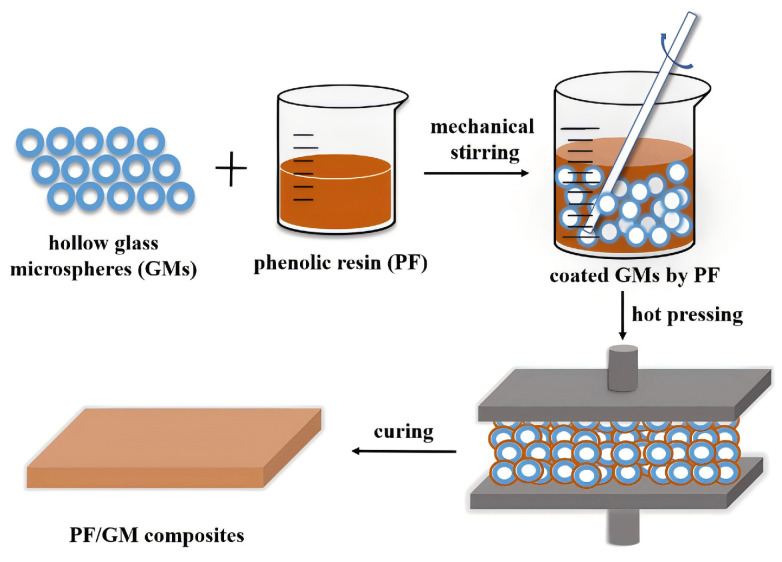
Schematic of the fabrication of PF/GM syntactic foams [98].

Wang [98] et al. prepared a phenolic matrix syntactic foam with a low density and good mechanical properties using phenolic resin as the matrix material and HGMS as a lightweight filler. The preparation process used in situ polymerization method and a thermos-compression molding process (see Figure 11). The experimental results showed that, as the microsphere content increased, both the compressive and tensile strengths of the composites decreased, whereas the modulus of elasticity increased. The composites were significantly better than the pure resin in terms of thermal stability and insulation, and the thermal conductivity of the synthesized foams was reduced by about 46.7% compared with that of the pristine resin when the content of microspheres was 20 wt.%. However, when the content of microspheres was too high, the mechanical properties of the composites decreased due to the fragmentation of the microspheres. Phenolic resin matrix synthetic foam is an ideal material for engineering applications; however, optimizing the filler content is crucial for achieving a balance between mechanical properties and density.

Phenolic resin exhibits low toughness and poor impact resistance. However, researchers have found that incorporating fillers, such as microspheres, can significantly enhance the impact resistance of phenolic resins. Huang [99] et al. filled different contents of surface-modified hollow glass beads into a phenolic resin matrix to prepare phenolic resin-based SBM, and the results of the study showed that the material possessed good mechanical properties, and the impact resistance was significantly improved. Additionally, it has good high-temperature resistance and can serve as a deep-sea high-temperature resistant material.

Phenolic resins offer significant structural and functional advantages as a buoyancy material matrix, but they also have some limitations. The rigid structure of phenolic resins may lead to higher material brittleness upon impact. In addition, phenolic resin processing typically requires high temperatures and pressures, which may increase production costs and process complexity. Hazardous substances can be released during the production and curing of phenolic resins, posing potential risks to the environment and human health. Despite these challenges, the high-performance characteristics of phenolic resins render them ideal matrix materials for specific applications. Through material design and modification, such as the addition of toughening agents or the use of more environmentally friendly production techniques, the overall performance of phenolic resin matrix buoyancy materials can be further enhanced to realize a wide range of applications in offshore engineering.

#### 3.2.2. Polyurethane (PU)

PU is a segmented copolymer consisting of rigid hard segments composed of chain extenders, and isocyanates cross-linked with flexible soft segments composed of polyethers or polyesters [100]. The unique structure of polyurethane’s soft and hard segments makes it possible to realize the modulation of the material’s hardness, strength, and other properties by changing the characteristics, such as the ratio of the soft and hard segments of the molecular chain and the degree of mixing [101]. Pellegrino [102] and others prepared HGMS/polyurethane matrix SBM with a density of 0.7 g/cm^3^, and the experimental results showed that the mixing structure of the soft and hard segments in polyurethane resins can effectively enhance the mechanical strength of PSBM.

**Figure 12:**
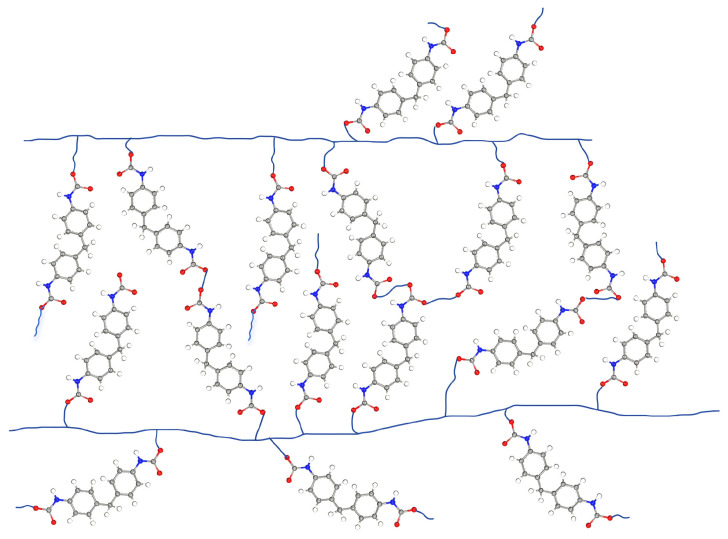
Schematic diagram of the molecular structure of flex-PUF [103].

Shu [103] et al. investigated a new type of flexible polyurethane foam (flex-PUF) with half-open and half-closed pores by adding HGMS with different volume fractions to improve its compressibility and energy absorption capacity, as shown in Figure 12, which illustrates the flowchart of the preparation (a) and the molecular structure (b) of flex-PUF. The effects of HGMS on the multiple impact protection and vibration damping properties of the material were analyzed through multiple impact tests and dynamic viscoelasticity experiments. The experimental results show that the flex-PUF filled with HGMS has a smaller maximum impact displacement and higher energy absorption efficiency under the same impact energy condition compared with the flex-PUF not filled with HGMS at the expense of some cushioning performance. The stiffness of the flex-PUF decreased with an increase in the number of impacts. In the vibration experiments, the viscous damping energy dissipation ratio of flex-PUF decreased with increasing frequency.

PU, as a matrix for buoyancy materials, offers a range of significant advantages and disadvantages. PU is known for its excellent chemical stability, mechanical properties, and abrasion resistance, which are properties that make it ideal for the manufacture of buoyancy materials. Their high elasticity and energy-absorbing capacity provide the necessary durability and impact resistance for marine floating structures. In addition, the low-temperature flexibility of PU and the network structure formed after curing give the material the advantage of maintaining its properties, even at extreme temperatures.

However, PU has a relatively high density, which may affect the buoyancy characteristics of the material. To overcome this disadvantage, it is often necessary to reduce the overall density by adding lightweight fillers, such as HGMS. In addition, the curing reaction of PU needs to be precisely controlled, and improper processing conditions may lead to the degradation of material properties. Environmental factors should not be overlooked either, as hazardous substances can be released during the production and curing of PU, posing potential risks to the environment and human health.

Despite these challenges, the overall performance of PU matrix buoyancy materials can be effectively enhanced through material design and engineering innovations such as optimizing formulations and curing processes. For example, the density and mechanical properties of PU can be balanced by adjusting its molecular structure and cross-linking density, and its processability and environmental friendliness can be improved by adding specific additives. Therefore, the application of PU in SBM is promising, especially in deep-sea applications that require high durability and reliability.

#### 3.2.3. Epoxy Resins (EP)

Epoxy resin is a general term for a class of polymers possessing two or more epoxy groups, which are stable, have excellent heat and corrosion resistance, have low water absorption, and are one of the most widely used polymer matrices for SBM [104]. Loubrieu [105] et al. used HGMS and three epoxy resin matrices with different glass transition temperatures to prepare PSBM. It was found that the strength of the EP matrix was sufficient to provide the PSBM with compressive strength under hydrostatic conditions, provided that the polymer matrix was well bonded to the interface of the HGMS. Qiao [106,107] et al. conducted extensive research on EP matrix lightweight and high-strength materials and developed a number of materials with adjustable density and high strength, as well as endowed with high-temperature resistance, which have the potential to be used in high-temperature and high-pressure environments in the deep sea.

**Figure 13:**
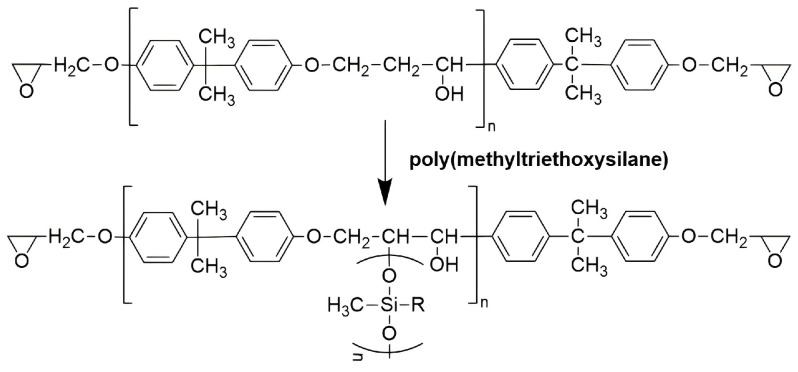
Schematic diagram of silicone-modified epoxy resin [108].

By blending reinforcing phases or grafting modified groups into the EP matrix, the mechanical properties thermal and chemical stability of the matrix are improved, which in turn improves the overall performance of SBM. He [36] et al. incorporated milled carbon fibers (MCF) into an EP matrix to increase the toughness by introducing an energy absorption mechanism. The results showed that when the weight ratio of MCF was 40%, the tensile modulus of the foam increased from 3.36 GPa to 5.41 GPa, and the fracture energy increased by 183% from 182 J/m^2^ to 516 J/m^2^. Liu [108] et al. prepared modified EP-HGMS SBM by chemically modifying epoxy resin using poly(methyltriethoxysilane) according to the schematic diagram shown in Figure 13 in order to improve the toughness of the material. The results of the study showed that the modification of the epoxy resin matrix resulted in a significant improvement in the performance indexes of the SBM in terms of density, water absorption, tensile strength, and compressive strength.

**Figure 14:**
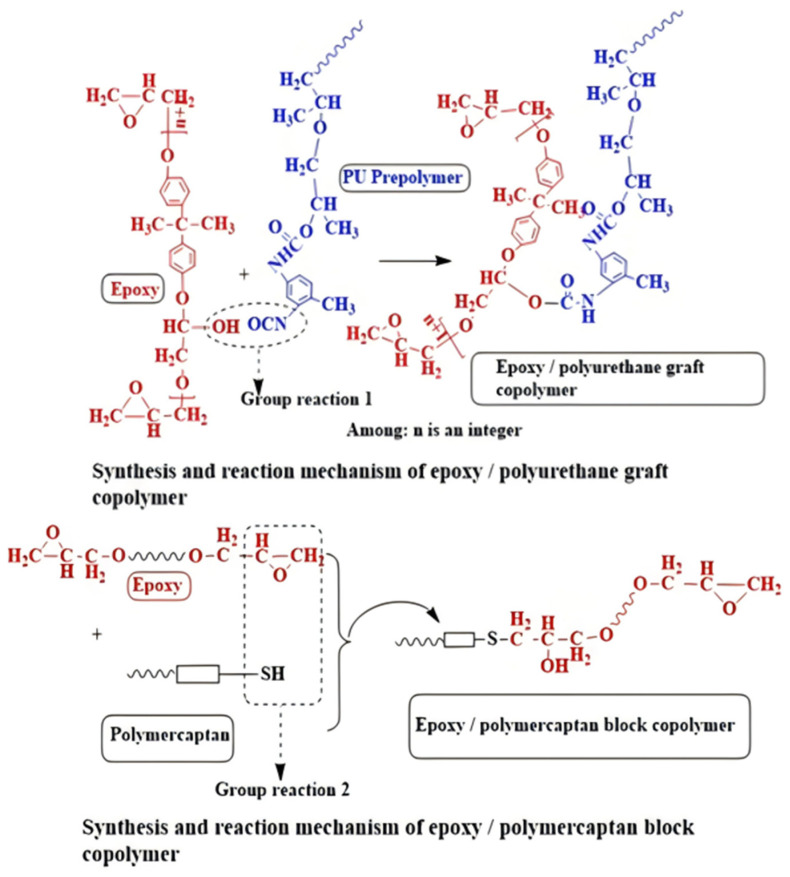
Schematic synthesis mechanism of the core matrix [109].

The grafting of modified groups onto the polymer matrix can impart different properties to the materials. Sun [109] et al. prepared a series of low-density composite syntactic foams using epoxy/polyurethane graft copolymer (E-g-U) and epoxy/poly mercaptan block copolymer (E-b-M) blended with varying amounts of HGMS(Figure 14 shows the schematic diagram of the main reaction flow). This type of material not only possesses excellent mechanical properties but also excellent damping properties, which are of high value for applications in deep-sea environments.

EP’s greatest strengths as a buoyancy material matrix are its excellent bonding properties and excellent chemical resistance, which allow it to bond effectively with other materials while resisting the corrosive substances found in the marine environment. EP’s thermal stability also allows it to maintain performance over a wide range of temperatures, which is critical for extreme temperature changes in the ocean. In addition, EP’s malleability allows the density and mechanical properties of PSBM to be customized by adjusting the formulation to suit different offshore engineering needs.

However, the disadvantages of EP matrix SBM should not be overlooked. Compared to phenolic resins, epoxy resins are slightly inferior in terms of heat resistance; compared to polyurethane, its flexibility and impact resistance are less impressive. In addition, epoxies cure at higher glass transition temperatures, which may affect their performance in certain applications requiring high elasticity. The shrinkage of epoxy resins during the curing process is also a consideration, as excessive shrinkage may lead to stress concentrations within the material, which may affect the integrity of PSBM.

In summary, while EP as a matrix is excellent in terms of adhesion, chemical resistance, and customizability, it needs to be further optimized and considered in terms of flexibility, heat resistance, and environmental impact.

#### 3.2.4. Curing Kinetics Study

The curing process has a significant effect on the properties of thermosetting resin. A proper curing process can ensure that the resin is fully cross-linked to form a stable three-dimensional network structure, thereby improving the mechanical properties of the material. The appropriate curing temperature and time are essential to avoid internal stress of the material, reduce holes and improve the bonding strength between the resin and the filler. Insufficient curing may result in reduced material properties such as reduced compressive strength and water resistance. However, overcuring can cause embrittlement of the material and affect its long-term stability. In addition, pressure control during curing is equally important for regulating the density and pore structure of the material, affecting the buoyancy characteristics and overall performance of the PSBM. Therefore, optimizing the curing process is a key step in achieving a high-performance thermosetting resin matrix, SBM.

The curing process involves the transformation of the thermosetting resin from a linear or branched molecular structure to a three-dimensional cross-networking structure. This process is achieved through a chemical cross-linking reaction, which plays a decisive role in the mechanical properties, thermal stability and chemical stability of the final material. A lot of work has been conducted on the curing process and curing kinetics of thermosetting resins. Yu [110,111] et al. studied the curing kinetic parameters and curing process of N,N,N,‘N‘-tetraepoxy-propyl-4,4′-diaminodiphenylmethane (AG-80) epoxy resin matrix SBM. The kinetic parameters of the curing reaction between AG-80 resin and m-XDA were calculated by DSC and non-isothermal thermal analysis (TAK). The study revealed that the apparent activation energy (Ea) decreased with an increase in the conversion. The experimental results show that when the volume fraction of HGMS is 55%, the density, uniaxial compression strength, saturated water absorption and water absorption of the composite are 0.668 g/cm^3^, 107.07 MPa, 0.17% and 0.025 h^−1/2^, respectively, indicating that the composite has the potential to be used as a deep-sea PSBM. In terms of the curing process, the process flow of pre-curing at 75 °C for 2 h, then curing at 90 °C for 2 h, and finally curing at 100 °C for 2 h was determined, which provided a scientific basis for the preparation of high-performance AG-80 epoxy resin matrix buoyancy material.

**Figure 15:**
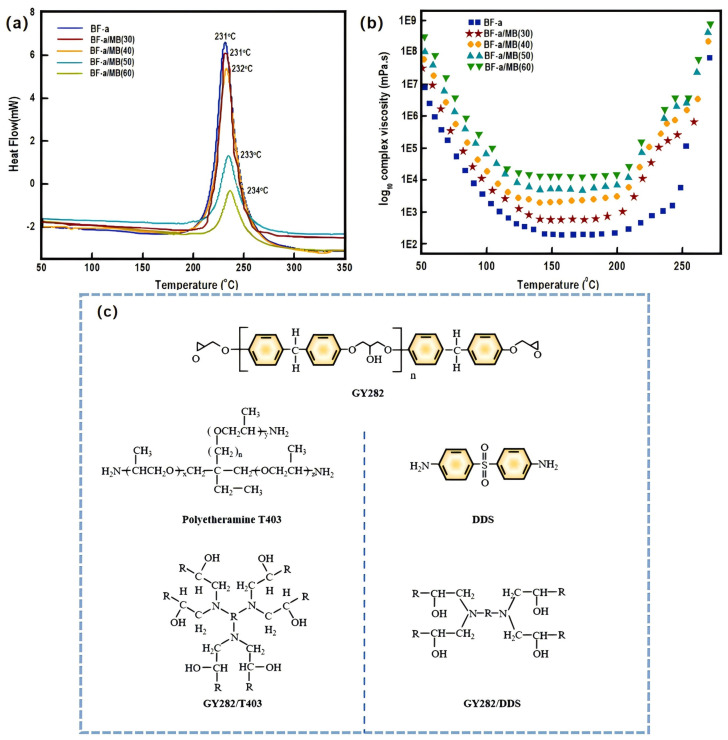
(**a**) Curing profile of BF-a/glass microsphere formulation [112]; (**b**) Viscosity–temperature curve of BF-a/HGM formulations [112]; (**c**) Chemical composition of GY282; T403; DDS; GY282/T403; GY282/DDS [113].

Gelation time is a key parameter in the curing process, which determines the point in time at which the resin transforms from a liquid to a solid network structure. The control of gelation time is crucial for the molding process. Ullas [112] et al. prepared thermally stable bisphenol F-based polybenzoxazine [poly(BF-a)] syntactic foams containing different volume fractions of HGMS. From the time versus cross-linking degree and viscosity curves shown in Figure 15a,b, the changing law of gelation time during the curing process was derived to provide a theoretical basis for optimal processing. Pei [113] et al. prepared two epoxy resin matrices with different properties using epoxy resin (GY282) and two flexible and rigid group curing agents, 4,4′-diaminodiphenyl sulfone (DDS) and Jeffamine T403, respectively(the structural formula of each reactive raw material is shown in Figure 15c). The effects of the resin matrix on the compression properties of the synthesized foams were systematically investigated. The interaction mechanism between the resin matrix and HGMS was discussed, and the failure mechanism was analyzed. The results of the study show that the selection of suitable curing agents can enhance the strength of SBM while strengthening the matrix.

Researchers have worked extensively on the curing process of thermosetting resin matrices for buoyancy materials in two dimensions, curing kinetics and curing process, and have achieved significant results. However, there are still shortcomings: first, the curing conditions, including temperature, time, and catalyst use, are able to modulate the crosslink density. Higher cross-linking densities typically enhance the rigidity and strength of a material, but excessive cross-linking may lead to brittle materials. The study of crosslink density can help stabilize the properties of the material in practical preparation applications. Second, volumetric shrinkage during curing affects the dimensional stability and internal stress state of the material, and research on how to control curing shrinkage is critical to ensure the long-term performance of buoyant materials.

## 4. Filler

Filler properties are a key factor affecting the performance of PSBM. HGMs have been a hot topic of research due to their low density, high strength and excellent chemical stability, and they are effective in reducing the overall density of the material while possessing high compressive strength and durability. Fly ash, an industrial by-product has also been explored as a filler to achieve the twin goals of cost-effectiveness and environmental friendliness. Hollow ceramic spheres reduce the density of the material and provide greater buoyancy. Hollow polymer microspheres are potential fillers for PSBM due to their adjustable physical properties and low cost. In addition, surface modification techniques for fillers, such as the use of silane coupling agents or polymer grafting, have significantly improved the interfacial bonding between the fillers and resin matrices, thereby improving the mechanical properties and water resistance of the composites. The thermal insulation properties of hollow fillers can effectively reduce the heat transfer of materials, and the establishment of a thermal conductivity model for hollow microbeads has great research significance for offshore oil development. These research advances not only promote the development of PSBM technology but also provide more optional high-performance materials for ocean engineering. In the following section, the applications of these fillers in PSBM are reviewed, and the effects of surface modification techniques on enhancing the material properties are discussed.

### 4.1. Hollow Glass Microspheres (HGMs)

HGMs are an indispensable lightweight filler for PSBM due to their remarkable properties of low density and high compressive strength. Their hollow structure significantly reduces the overall density of the material, providing buoyancy that makes them particularly suitable in high-pressure environments, such as deep-sea exploration and offshore engineering. The chemical stability and corrosion resistance of HGMS further enhance its reliability in marine applications. This section mainly focuses on the application of HGMS in PSBM and discusses how it can be finely tuned to optimize the material properties to meet the stringent requirements of buoyancy and durability in deep-sea operations.

#### 4.1.1. Effect of HGMS Volume Fraction on Material Properties

**Figure 16:**
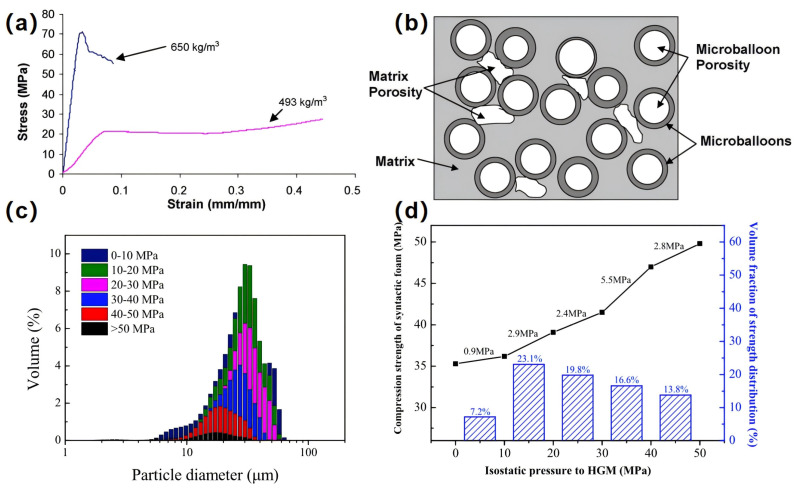
(**a**) Compressive stress–strain curves for plain syntactic foams having different densities [114]; (**b**) Schematic representation of the syntactic foam structure showing microspheres and matrix porosities [114]; (**c**) Particle size distribution of strength distribution of a batch of T30 HGM [55]; (**d**) The relationships of relative increment of SF-T30-P compression strength and the volume fraction with strength distribution of T30 HGMS [55].

In the field of marine applications, compressive strength and density are two important indicators for the use of PSBM, and how to minimize the density of the material while meeting the compressive strength is still a key issue to be addressed in the development of high-performance PSBM. The way to reduce the density is mainly by adding HGMS to the polymer matrix, and most PSBM reduce the density by increasing the content of HGMS, thus realizing the provision of greater buoyancy. However, the results of Gupta [114] and others show that the compressive strength of SBM tends to decrease with decreasing density (see Figure 16a). The reason for this is, on the one hand, that the strength is not as strong as the increased volume fraction of HGMS in the matrix, which reduces the strength of the material. On the other hand, the reduction of density is accompanied by the appearance of excessive matrix pores in the matrix resin, as shown in Figure 16b between the matrix and the HGMS because of the residual air, the presence of matrix pores, which reduces the strength of the composite and can lead to an increase in moisture absorption. Zihlif [115] et al. investigated the mechanical properties of HGMS/EP two-component SBM and found that the mechanical property indexes such as material density, modulus of elasticity, compressive yield stress, and yield strain of this two-component SBM decrease with an increase in the HGMS volume fraction.

With the continuous development of computer technology, advanced software techniques have been used to deal with the relationship between the filler volume fraction and the performance of PSBM. Chen [54] et al. used ANSYS finite element software for the simulation of the full ocean depth buoyancy material composed of HGMS and epoxy resin to establish a body-centered cubic cell fine mechanics model and obtained the relationship between the volume fraction of HGMS, the material effective volume fraction of HGMS, effective modulus of elasticity and specific gravity of the material, which provides a theoretical basis for the development of high-performance full-depth buoyancy materials.

#### 4.1.2. Influence of HGMS Properties on Material Properties

The strength of HGMS itself also plays an extremely important role in the performance of the material. The strength of an HGMS is often related to its wall thickness, particle size, and product material.

Wang [55] et al. distinguished HGMS with different compressive strengths by treating them under different hydrostatic conditions to prepare the corresponding syntactic foams. From Figure 16c, we can see that HGMS in the smaller particle size range exhibits higher compressive strength percentages. As the particle size increases, the compressive strength percentage decreases, which indicates that smaller particle size HGMS tend to exhibit higher strength. Figure 16d depicts the relationship between the relative increment of the compressive strength of the material and the compressive strength and volume fraction of the HGMS. When the compressive strength of HGMS is below 30 MPa, the increase in compressive strength of the syntactic foams is closely related to the volume fraction, which may be due to the fact that the compressive failure damage of the syntactic foams is mainly caused by the extrusion rupture of HGMS. When the compressive strength of the HGMS increased, the compressive strength of the composite foam increased accordingly. When the strength of HGMS is between 30 and 40 MPa, there is a clear inflection point in the compressive strength of the syntactic foams, and the failure is no longer caused by the extrusion rupture of HGMS. Based on SEM images, Wang’s study demonstrated that the damage to the syntactic foam was mainly caused by peeling the epoxy resin matrix.

Swetha [116] et al. prepared an EP matrix SBM with different HGMS volume fractions and wall thicknesses. It was found that the strength and modulus decreased with increasing HGMs volume fraction and increased with increasing HGMS wall thickness. SBM in service in the marine environment is prone to changes in water depth and sudden changes in water pressure. Therefore, Rousseau [117] et al. prepared a different HGMS/EP matrix SBM and investigated the relationship between the type of HGMS and the impact resistance performance under static and dynamic pressures and the influence of the particle size of the HGMs on the impact performance, respectively. The results show that, statically, HGMs with a small particle size and large wall thickness gives the material greater compressive strength, but dynamically, larger-sized HGMS have a better cushioning effect.

Summarizing the above studies, it can be seen that the wall thickness of HGMS has a significant influence on mechanical properties such as compressive properties and fracture characteristics of PSBM. The larger the wall thickness of the HGMS with greater compressive strength, the greater the compressive role played in the PSBM. Therefore, the selection of HGMS with a greater wall thickness is a feasible way to increase the compressive strength of the PSBM. However, a greater wall thickness often implies a greater density.

#### 4.1.3. Study on Processing Integrity of HGMS in Thermoplastic Resins

HGMS are widely used to reduce material density due to their low density and high compressive strength, but their fragility during processing requires special attention. A range of strategies can be adopted to improve the integrity of HGMS in the process.

First, a twin-screw extruder (TSE) that rotates in the same direction is recommended for mixing HGMS with the polymer to achieve a continuous and gentle process. Secondly, the point of introduction of HGMS should be optimized downstream of the extruder, and the polymer should be added in a fully molten state through the side feed or top feed port, thereby reducing the shear stress on the microbeads. In addition, the use of a high free volume conveying element reduces the shear rate by increasing the depth of the screw channel, which is particularly important for protecting low-density HGMS. At the same time, the preheating of HGMS helps to maintain the viscosity stability of the polymer melt. During the pelletizing process, the use of underwater pelletizing machines significantly reduced the breakdown of HGMS, while the selection of resins with low viscosity and high melt flow index (MFI), as well as softer and more elastic polymer substrates, further reduced the risk of damage to HGMS during mixing. The use of surface treatment technology can further improve the dispersion of HGMS and its compatibility with the substrate to achieve the efficient application of HGMS in thermoplastic resins without sacrificing performance. The combined application of these strategies provides a solid foundation for the integration of HGMS into lightweight and high-performance composites [118].

### 4.2. Fly Ash

Fly ash, an innovative filler for PSBM originating as a by-product of coal-fired power plants, has gained attention for its unique structure and properties. This finely powdered material, composed primarily of silicates and aluminates, is porous, has a high specific surface area and spherical particle morphology, and its density is typically lower than that of conventional fillers. These properties of fly ash give it excellent flowability and dispersibility, helping to reduce the overall density of the material while maintaining some mechanical strength. In addition, the high porosity and thermal insulating properties of fly ash provide additional environmental resilience and durability to the PSBM. The application of fly ash in PSBM not only realizes the high-value utilization of industrial wastes but also demonstrates its potential in marine engineering due to its cost-effectiveness and environmental friendliness.

Bharath Kumar [61] et al. investigated syntactic foams using high-density polyethylene as matrix material and fly ash as lightweight filler. The experimental results showed that as the amount of fly ash filler increases, the density of the material decreases, and the modulus increases, but the strength decreases. Bharath Kumar also estimated the properties of fly ash through theoretical models in order to provide a theoretical basis for the design and optimization of syntactic foams. The use of fly ash not only improves the buoyancy properties of the material but also helps in the environmentally friendly utilization of industrial waste.

**Figure 17:**
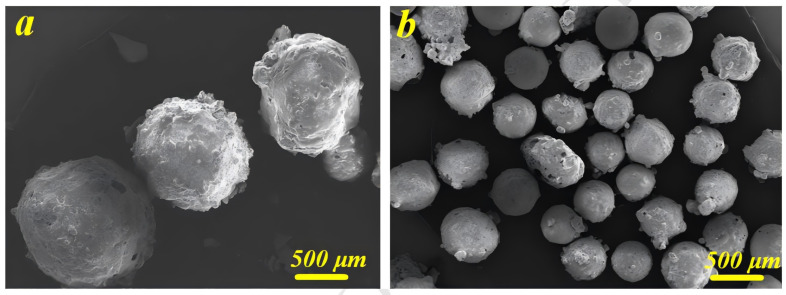
Microstructure of two hollow spheres for composite foams [60]. (**a**) Hollow microspheres with average diameter and wall thickness of 950 ± 200 μm and 50 ± 10 μm, respectively; (**b**) Hollow microspheres with average diameter and wall thickness of 450 ± 200 μm and 30 ± 10 μm, respectively.

Fan [60] et al. explored the dynamic compressive behavior of syntactic foams prepared with fly ash as a lightweight filler in combination with polyurethane foam (PUR). Syntactic foams prepared by pressure impregnation method utilizing different sizes of fly ash (Figure 17) were investigated for the effect of its size and internal lateral constraints on the dynamic properties of the material. The experimental results showed that the large-size fly ash-filled syntactic foams showed an increase in dynamic compressive strength of 35% to 77% compared to quasi-static compression, but the high stresses were insensitive to the strain rate. The introduction of the aluminum honeycomb structure significantly increased the mechanical response and energy absorption of the syntactic foams while improving the dynamic response of the material. The reuse of fly ash as an industrial waste material not only reduces the weight of the material but also improves its mechanical properties. However, the strain-rate sensitivity of the material and the shift in damage modes pose new challenges for design and application.

Fly ash is an ideal lightweight filler in PSBMs due to its low density, high compressive strength and good chemical stability. The hollow structure of fly ash helps reduce the overall material density while maintaining the necessary mechanical strength to provide the buoyancy required in marine engineering, such as deep-sea exploration. The reuse of fly ash is also in line with environmental and sustainability trends, helping to reduce the environmental impact of industrial waste.

However, there are some limitations of fly ash as a filler. Firstly, their physical properties, such as wall thickness and size distribution, affect the final properties of the composites. Smaller wall thicknesses may lead to fragmentation of fly ash during processing, which can affect the mechanical properties and durability of the buoyancy material. Secondly, the surface treatment of the fly ash and its compatibility with the matrix resin are also critical factors that need to be improved by surface modification or the use of coupling agents to enhance its adhesion strength with the resin matrix in order to optimize the performance of the composites.

Combined with the above studies in the literature, fly ash shows great potential for improving the buoyancy and mechanical properties of PSBM. However, it is also necessary to consider its dispersion in the material, fragmentation during processing and compatibility with the matrix resin. By optimizing these factors, the advantages of fly ash can be fully utilized, and its limitations can be overcome, leading to the development of PSBM with better performance.

### 4.3. Hollow Ceramic Spheres

Hollow ceramic spheres are used as fillers for SBM in order to further reduce the overall density of the material and to meet the lightweight requirements of deep-sea exploration and ocean engineering equipment. Although conventional two-component PSBM can reduce the material density by increasing the content of hollow microspheres, there exists a limit beyond which the material properties may be degraded. The three-component PSBM prepared by adding large-sized hollow ceramic spheres not only has a lower density but also excellent compressive strength and stability, which is especially suitable for applications in deep-sea environments. However, the implosion problem that large-sized hollow spheres may face in high-pressure environments introduces new challenges for material design and fabrication. In this section, the application of hollow ceramic spheres in PSBM is described.

**Figure 18:**
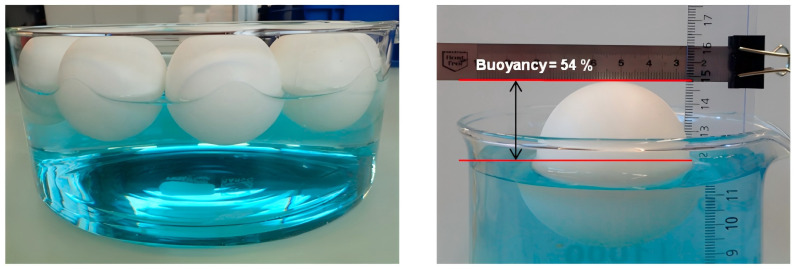
Buoyancy of sintered spheres in simulated seawater [120].

Shchegoleva [119] et al. provided an in-depth analysis of alumina-based ceramic spheres as fillers for deep-sea buoyancy modules to replace traditional synthetic foams containing glass microspheres. The performance of the ceramic spheres was tested experimentally at pressures corresponding to different water depths, and the stability and effectiveness of the hollow ceramic spheres were analyzed by strength assessment. It was shown that hollow ceramic spheres exhibit potential as SBM due to their low density, high compressive strength and excellent pressure resistance. However, the fabrication of seamless and ideally shaped ceramic spheres, as well as life tests with long-term or cyclic loading at different depths, remain challenges. Rastogi [120] et al. presented an innovative production method for hollow ceramic spheres for deep-sea applications. With this technique, alumina-based ceramic spheres with a diameter of 52.8 mm, wall thickness of 1.08 mm, and density of 0.46 g/cm^3^ were successfully fabricated (see Figure 18) with a buoyancy of 54%. These spheres exhibited extremely high hydrostatic collapse pressures in excess of 200 MPa after sintering at 1630 °C. Compared with conventional gypsum molds, pulp molds offer better structural integrity in the wet state, avoid manual demolding steps, and simplify the production process to improve productivity.

SBM with added hollow ceramic spheres possess low density, high strength, and excellent pressure resistance, as well as thermal and chemical stability, making them more reliable and durable in marine environments.

However, the application of hollow ceramic spheres faces some challenges. The brittle nature of ceramic materials may create a risk of implosion in deep-sea high-pressure environments, which requires precise design and testing of the structural integrity and pressure resistance of the ceramic spheres. In addition, the high cost of manufacturing ceramic spheres and the complexity of their production process limit their widespread application. The geometric accuracy and surface quality of ceramic spheres have a significant impact on their performance, and fine manufacturing processes are required to ensure their reliability in deep-sea environments.

Despite these challenges, the application of hollow ceramic spheres shows great potential, especially in ocean engineering and exploration operations in medium to deep waters. Through continuous optimization of the material design and manufacturing processes, hollow ceramic spheres are expected to further improve the performance of SBM and meet the needs of future deep-sea exploration.

### 4.4. Hollow Polymer Microspheres (HPMs)

In order to solve the problem of interface properties between the filler and polymer matrix, researchers have tried to use organic hollow microspheres as the buoyancy-regulating medium of PSBM. Hollow Polymer Microspheres (HPMS) are a new type of hollow microsphere. Compared with HGMs, HPMs have the advantages of low density, good toughness, and good compatibility with the resin matrix [121,122,123]. The elastic properties of HPMS can absorb and disperse energy when impacted, thereby improving the impact resistance of the material. HPMS usually has good corrosion resistance and hydrolysis resistance, which helps to improve the stability of the material in harsh environments. Xie [124] et al. used HPMS with a particle size of about 25 μm and a packing density of 0.08–0.10 g/cm^3^ and epoxy resin CYD-127 to prepare syntactic foams with a density of 400–500 kg/m^3^ and a strength of 10–20 MPa. It has the potential to be used as buoyancy material in marine negative-pressure environments.

However, the mechanical properties of HPMS are not as good as those of inorganic material microbeads such as HGMS. Dando [125] et al. prepared PSBM containing thermoplastic HPMS with 75–95 vol% of microspheres in the composites and composite densities of 0.067–0.43 g/cm^3^, but with low compression strength <15 MPa. Although the use of low-density thermoplastic HPMS reduces the density of the material, its strength is not high, and the material has more internal defects and lower strength, which limits its application in the deep-sea field.

**Figure 19:**
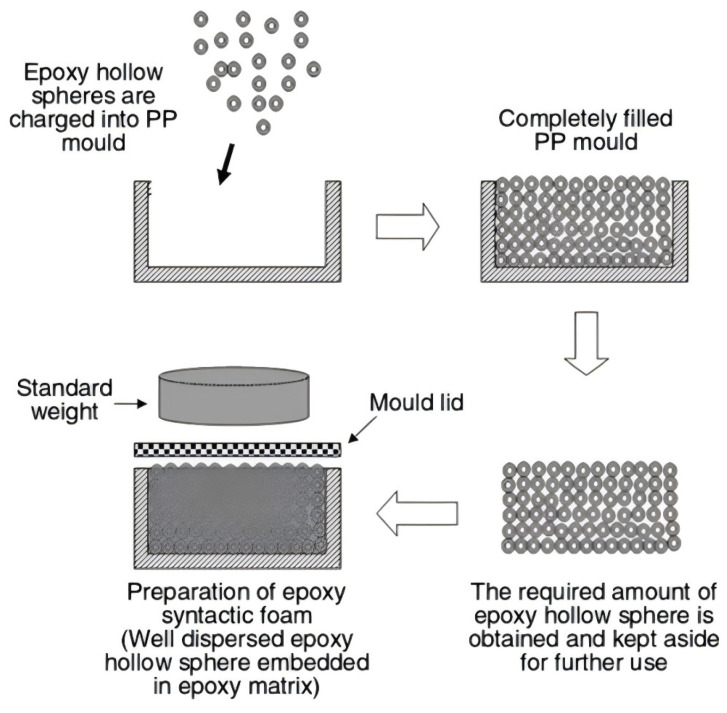
The summary of the initial preparation step and the production of a well dispersed sphere within the mold [127].

In addition, expandable microspheres are a new type of HPMS, which is different from traditional chemical foaming materials in that its foaming and expansion processes have the advantages of controllable speed and controllable size. Therefore, expandable microspheres can avoid uncontrollable factors in the expansion process, and buoyant materials prepared by adding expandable microspheres have a larger density and compressive strength distribution. Wang [126] et al. prepared PSBM using expandable microspheres and epoxy resin with a density of 450–800 kg/m^3^ and a compressive strength of 15–50 MPa for a wide range of applications and a superior preparation method. Samsudin [127] et al. prepared epoxy hollow spheres by coating the surface of expanded polystyrene (EPS) bead pellets with epoxy resin and step high-temperature curing to shrink all the EPS beads inside the epoxy-coated spheres, creating a hollow structure inside the spheres. The epoxy hollow spheres were mixed into the epoxy resin matrix to prepare syntactic foams (the preparation process is shown in Figure 19), which has the advantage of low cost and provides an effective way to improve the interfacial compatibility of syntactic foams.

**Figure 20:**
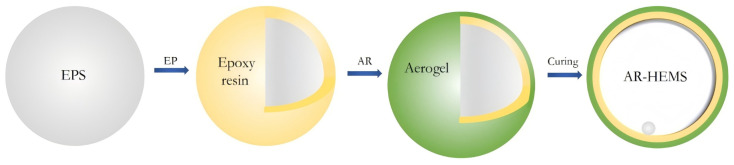
The schematic diagram of the preparation of the AR-HEMS [128].

Wu [128] et al. utilized the characteristics of ultra-low density and good impact resistance of aerogel to prepare lightweight aerogel-reinforced hollow epoxy microspheres (AR-HEMS) using aerogel material, epoxy curing agent system and expandable polystyrene bead grains (EPS). The surface of the EPS bead grains was first covered with an epoxy curing agent system, and a sufficient amount of aerogel powder was sprayed so that the aerogel powder was completely wrapped around the EPS beads. A layer of AR-HEMS was prepared after the epoxy on the surface of the EPS bead grains had been completely cured. By repeating the above steps once or twice, two and three layers of AR-HEMS can be prepared. A schematic diagram of the preparation of AR-HEMS is shown in Figure 20. HPMS-EP SBM was prepared by compression molding method. The results show that the material has a density of 0.428 g/cm^3^ and a compressive strength of 20.76 MPa, and it can be applied in the deep sea at 2000 m. The material is a good choice for the deep-sea application.

The incorporation of HPMS as a filler into SBM provides tunable properties and environmental stability and plays a significant role in increasing interfacial compatibility, reducing density, and improving impact properties. However, the high cost of synthesizing HPMS, especially when using special polymers or complex manufacturing processes, and the challenge of maintaining the hollow structure of the microbeads from rupturing during processing requires fine control of the processing conditions. Most critically, the mechanical properties of HPMS can limit the use of PSBM in certain high-loading applications.

Combined with the current status of HPMS research, this paper argues that future research should focus on the development of high-performance HPMS, the study of interfacial interaction mechanisms, the design of microbead structures, multiscale simulation, and the integration of functions such as sensing and self-healing.

### 4.5. Surface Modification of Hollow Microspheres

Surface modification of inorganic hollow microspheres can significantly improve the adhesion and stability between the hollow microspheres and the resin matrix and avoid the aggregation of fillers in the matrix, thus enhancing the mechanical properties of the materials. There are two ways to enhance the performance of PSBM. On the one hand, functional fillers are grafted on the surface of inorganic hollow microspheres or chemically modified using coupling agents. On the other hand, physical surface treatments, such as acid and heat treatments, can be applied to the inorganic hollow microspheres to enhance the bonding with the polymer matrix.

**Figure 21:**
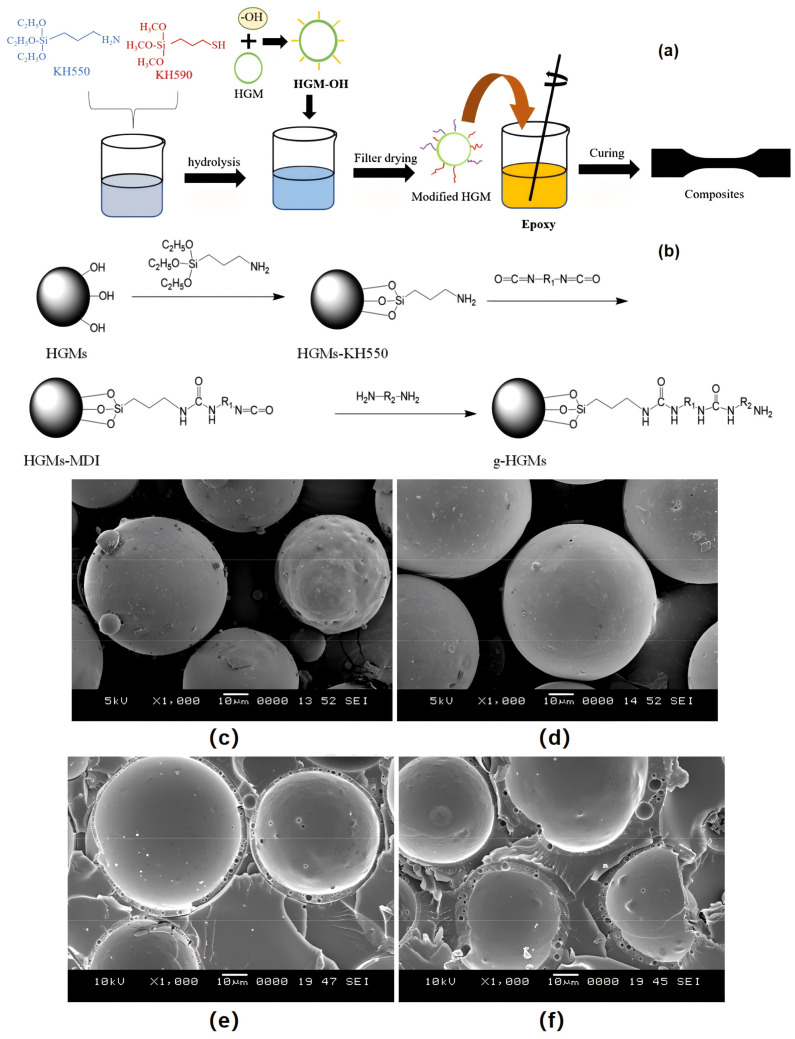
(**a**) Preparation process of the compound coupling agent-modified HGM/EP composites [132]; (**b**) Schematic preparation of the hollow glass microspheres grafted with the curing agent of epoxy [131]; (**c**) Unmodified hollow microbeads [130]; (**d**) Modified hollow microbeads [130]; (**e**) Unmodified hollow beads have poor bonding with matrix [130]; (**f**) Modified hollow beads bonded well with the matrix [130].

Shahapurkar [129] et al. prepared epoxy resin matrix syntactic foams using silane-treated HGMS. The results showed that silane-treated HGMS improved the erosion resistance of the syntactic foams. Doddamani [130] et al. used 3-amino propyl triethoxy silane (APTS) for the surface modification of fly ash to prepare syntactic foams. Figure 21c–f shows the SEM images of fly ash before and after surface modification and their bonding to the matrix; from the images, it can be seen that the surface-modified fly ash microbeads are more tightly bonded to the matrix. The results showed that surface treatment improved the modulus of elasticity and compressive strength of the syntactic foams. Li [131] et al. prepared syntactic foams by incorporating HGMS grafted with an epoxy resin curing agent into an epoxy resin matrix. The surface modification process of HGMS is shown in Figure 21b, where NaOH caused hydroxyl groups to form on the surface of HGMS, which were grafted with the amine-based end-capping coupling agent KH550, curing agent MDI, and low molecular weight polyamide 651. The results of the study showed that HGMS grafted with an epoxy curing agent enhanced the syntactic foams.

Zhu [132] et al. used two silane coupling agents as composite coupling agents to prepare composite coupling agent-modified HGMS/EP syntactic foams (the preparation flow chart is shown in Figure 21a). The results showed that the composite coupling agent could effectively improve the interfacial bonding between HGMS and matrix resin. The voids and pores between the modified HGMS and the matrix resin almost disappeared. The composite coupling agent-treated materials exhibited excellent mechanical properties, along with lower density and lower water absorption. Compared with the composites without HGMS modification, the compressive strength, tensile strength, tensile modulus, flexural strength, and flexural modulus increased by 51.43%, 62.23%, 65.51%, and 32.26%, respectively, after modification.

Zhang [133] et al. used aminopropyltriethoxysilane coupling agent to modify the surface of HGMs, a more uniform transition layer was formed between HGMs and epoxy resin, and the compression strength of PSBM was significantly improved. When the volume fraction of HGMs was 60 vol%, the compression strength increased from 62.7 MPa to 77.3 MPa, which was 23.33% higher. Yuan [134] et al. showed that quenching the surface of HGMs resulted in a decrease in the content of alkali metal oxides and an increase in the hydrophobicity of the HGMs surface, and the synergistic effect of these two factors led to an increase in the affinity between the polymer matrix and HGMs. The interfacial adhesion between the microsphere surface and the matrix resin was enhanced, and the compressive strength and fracture toughness of the material were significantly increased.

The synthesis of previous research results shows that the surface modification of hollow microspheres plays a crucial role in enhancing the comprehensive performance aspects of PSBM. The surface modification strategy significantly enhanced the mechanical behavior of the materials by enhancing the adhesion strength between the hollow microspheres and the resin matrix. In addition, surface modification effectively reduced the aggregation tendency of the hollow microspheres in the matrix and promoted their uniform dispersion in the matrix, thus enhancing the homogeneity and stability of the materials. Suitable surface modification also conferred additional chemical stability to the hollow microspheres, improving their resistance to environmental factors, such as moisture and chemical attack. At the same time, modulating the surface energy of the hollow microspheres through surface modification optimizes their interaction with the matrix, which in turn improves the physical properties of the material, including lowering water absorption and increasing pressure resistance.

However, the surface modification process requires precise control of treatment conditions, including temperature, time, and modifier concentration, which increases the complexity of the preparation process. In addition, some modifiers may pose potential risks to the environment. Therefore, future research should focus on developing environmentally friendly modifiers to achieve sustainable development goals that are cost-effective and have low environmental impact. At the same time, the application of nanotechnology in the surface modification of hollow microspheres should be explored to achieve finer interface control. The interfacial dynamics between the hollow microspheres and the matrix materials were studied to reveal the deep mechanism of interfacial bonding.

### 4.6. Study on Thermal Insulation Performance of HGMS Composite Buoyancy Material

SBM plays a vital role in the field of offshore oil development. Offshore oil transport pipelines need to have good buoyancy characteristics to maintain stability. Due to their low-density properties, hollow microspheres are able to effectively reduce the overall weight of the material while providing the necessary buoyancy, which is essential for deep-sea oil exploration and production. At the same time, hollow microspheres have low thermal conductivity due to their internal cavity structure. When these microspheres are filled into SBM, the overall thermal conductivity of the material can be effectively reduced, thus reducing the transfer of heat. This feature prevents the formation of waxy or ice crystals during the long-distance transportation of crude oil in cold sea water, which precipitates on the oil pipeline shell and causes blockage accidents. Based on the thermal insulation characteristics of the material, the establishment of a thermal conductivity model for hollow microspheres is of great significance for offshore oil development.

**Figure 22:**
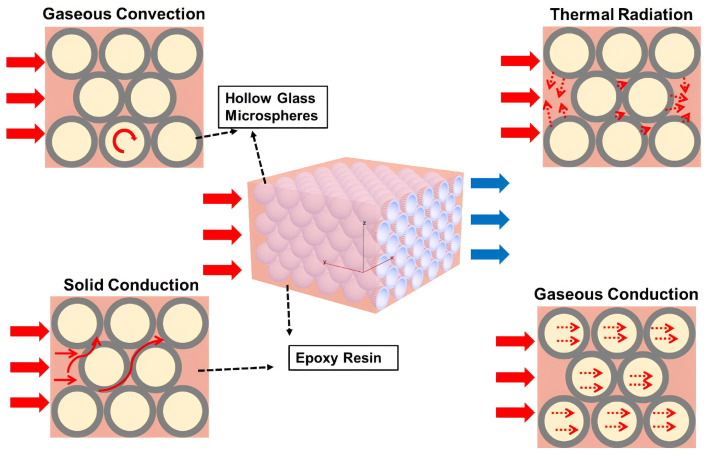
Schematic of thermal transport pathways in composite material [136].

Liao [135] et al. analyzed in detail the thermophysical properties of silica hollow microspheres. Through experimental tests, theoretical formula calculations and finite element simulations, it is found that the thermal conductivity of these microspheres is lower than 0.02 W/(m·K), indicating that they have excellent thermal insulation performance. The effects of the particle size and packing density on the thermal conductivity were also observed, and the heat transfer characteristics of the hollow microspheres were further revealed by finite element analysis. Xing [136] et al. prepared a lightweight insulation material suitable for offshore oil pipelines with a density of only 0.591 g/cm³ and whose bending strength and modulus can reach 22.34 MPa and 1.34 GPa, respectively. The researchers constructed a three-phase prediction model to predict the material’s effective thermal conductivity. The accuracy of the model is verified by comparing the experimental results with the calculated values from the three-phase prediction model. The results show that the thermal conductivity of the composite decreases significantly with an increase in the particle size and volume fraction of HGMS. As shown in Figure 22, there are three main ways of heat transfer in composite materials: (l) gas convection in HGMS, (2) Thermal radiation of HGMS surface; and (3) solid and gas conduction. When heat is transferred to the EP matrix of the composite material, part of the heat is transferred by the EP, and the other part is transferred by the HGMS. Due to the low thermal conductivity of HGMS, the heat conduction path of HGM-filled composites is longer, the heat barrier is larger, and the heat insulation performance is better.

In addition to epoxy resin matrix composites, the researchers also added hollow microspheres to different resin matrices and studied their thermal conductivity. Patankar [137] et al. discussed the thermal and mechanical properties of composites filled with HGMS using high-density polyethylene (HDPE) as the matrix. A new lightweight thermal insulation material with customized thermal conductivity and mechanical properties by adjusting the HGMS content has been developed. Liang [138] et al. used the thermal conductivity equation to estimate the thermal conductivity of PP composites filled with HGMS of different sizes. Compared with the measured data, the predicted thermal conductivity is in good agreement with the measured data. The results show that the addition of HGMS has a significant effect on the thermal properties of PP composites, the thermal conductivity decreases linearly with an increase in the HGMS volume fraction, and the particle size of HGMS has little effect on the thermal conductivity of the PP/HGMS composites.

Hollow microspheres have shown significant application potential in the field of thermal insulation materials due to their lightweight characteristics and low thermal conductivity. Effectively dispersing the hollow microspheres in different polymer substrates not only reduces the weight of the material but also significantly improves the thermal insulation performance. This study reveals the important effects of the particle size and volume fraction of hollow microspheres on the thermal conductivity of composites and shows the possibility of optimizing the thermal insulation performance by adjusting these parameters. The combination of mathematical model and experimental verification provides a reliable method for predicting the thermal conductivity of hollow microspheres filled composites. These advances provide a solid theoretical basis, and experimental data support the development of new lightweight materials to meet specific thermal insulation needs, especially in deep-sea oil development, building energy conservation, and other applications, showing great application potential and development prospects.

## 5. Conclusions and Outlook

As a new functional material, PSBM has made remarkable progress in the field of marine exploitation. Due to its excellent strength, water absorption and low-density characteristics, it has attracted wide attention. Based on their composition and manufacturing process, PSBM can be categorized into single-component, dual-component, and triple-component types, each suited for different operational depths. In recent years, a lot of research has been conducted on the preparation process, performance testing and model simulation of PSBM, and a lot of research results have been obtained. A complete range of related products can meet most of the current use scenarios.

The performance of PSBM is closely related to two factors: the matrix and filler. This paper briefly summarizes and discusses the structure, performance, and process of PSBM. Focusing on the two dimensions of the matrix and filler, research on PSBM at home and abroad has been conducted in recent years. Despite the progress above, there is still space for improvement. This paper suggests that in-depth research can be carried out in the following aspects:

(a) As an important component of the PSBM, the performance of the resin matrix directly affects the overall performance of the PSBM. The strength and toughness of the resin matrix can be enhanced by chemical modification and the addition of a reinforcing phase to meet the needs of ultra-deep sea applications. Meanwhile, in the military field, damping performance and acoustic performance are of great significance for PSBM, and the current research in this area is still relatively lacking; further research is needed to provide the resin matrix with these special properties. In some areas of the seabed (such as submarine volcanic vents), high temperature is also an important condition limiting the performance of PSBM, and the development of materials with high-temperature-resistant properties also needs to be explored in the preparation of resin matrixes. In the preparation process, the coordination of foaming, curing, and other steps with filler mixing also requires specialized and in-depth research to achieve high-performance products on the ground.
(b) Hollow microspheres and other fillers, because of their unique hollow structure, can reduce the density of the material at the same time, provide a certain degree of strength and stability. However, most of the current hollow microspheres are much weaker than the resin matrix, and the performance of the filler has become the shortboard of the overall performance of PSBM and the development of high-performance hollow beads is of key significance for improving the performance of the product. At the same time, more in-depth research is needed to clarify the influence of the filler parameters and the preparation process on the performance of PSBM. This may require the application of more computer simulation technology, but unfortunately, the current simulation software is mostly applied to the failure analysis of PSBM and less to provide theoretical support for the preparation of materials.
(c) The combination of the filler and matrix is an extremely critical factor affecting the performance of PSBM. Research on surface modification techniques of inorganic hollow microspheres, such as environmentally friendly modifiers, nano-modification techniques, fine interfacial control, and interfacial dynamics studies, is of great significance for improving interfacial compatibility. Meanwhile, high-performance HPMS should be developed and endowed with integrated functions such as sensing and self-healing.

## Figures and Tables

**Table 1:** Influence of stacking volume fraction of GR-HEMS on compressive properties and density of three-component PSBM [50].

Stacking Volume Fraction of GR-HEMS	0	20	40	60	80
Compressive Strength (MPa)	61.4	41.6	29.4	23.1	21.4
Density (g/cm^3^)	0.676	0.659	0.632	0.602	0.573

**Table 2:** Influence of wall thickness of GR-HEMS on compressive strength and density of three-component PSBM [50].

Wall Thickness of GR-HEMS	1	2	3
Compressive Strength (MPa)	16.0	21.0	25.2
Density (g/cm^3^)	0.471	0.563	0.614

**Table 3:** Performance of the polymer matrices.

Polymer Matrix	Density (g/cm^3^)	Tensile Strength (MPa)	Flexural Modulus (GPa)	Elongation at Break (%).	Water Absorption (%, 24 h)
Thermoplastic resin					
HDPE	0.95–0.97	20–40	0.8–1.5	100–550	0.01
LDPE	0.92–0.93	8–30	0.25–0.35	100–650	0.01
PP	0.90–0.91	30–40	0.35–1.5	50–400	0.01
PA	1.0–1.15	60–75	1.07–2.32	20–500	0.04–4
EA	1.0–1.2	60–90	3.1–3.5	100–800	1.5
PMMA	1.15–1.19	50–80	2.5–3.5	2–15	0.1–0.3
Thermosetting resin					
Phenolic resin	1.7–2.0	50–125	8–23	<1	0.01–1.2
Epoxy resin	1.1–1.4	35–140	14–30	<5	0.03–0.2
Polyurethane	1.0–1.1	70	4	3–6	0.02–1.5

## Data Availability

No new data were created or analyzed in this study.
